# Oxidative Stress in the Pathophysiology of Chronic Venous Disease: An Overview

**DOI:** 10.3390/antiox14080989

**Published:** 2025-08-12

**Authors:** Sonia Rațiu, Mihaela I. Mariș, Adina V. Furdui-Lința, Laurențiu V. Sima, Tiberiu I. Bratu, Adrian Sturza, Danina M. Muntean, Octavian M. Crețu

**Affiliations:** 1 Doctoral School Medicine, “Victor Babeș” University of Medicine and Pharmacy of Timișoara, Eftimie Murgu Sq. No. 2, RO-300041 Timișoara, Romania; ratiu.sonia@umft.ro (S.R.); adina.linta@umft.ro (A.V.F.-L.); ion.bratu@umft.ro (T.I.B.); 2 Center for Translational Research and Systems Medicine, “Victor Babeș” University of Medicine and Pharmacy of Timișoara, Eftimie Murgu Sq. No. 2, RO-300041 Timișoara, Romania; maris.ioana@umft.ro; 3 Department Functional Sciences-Pathophysiology, “Victor Babeș” University of Medicine and Pharmacy of Timișoara, Eftimie Murgu Sq. No. 2, RO-300041 Timișoara, Romania; 4 Department of Surgery I—First Clinic of Surgical Semiology & Thoracic Surgery, Center for Hepato-Biliary and Pancreatic Surgery, “Victor Babeș” University of Medicine and Pharmacy of Timișoara, Eftimie Murgu Sq. No. 2, RO-300041 Timișoara, Romania; sima.laurentiu@umft.ro (L.V.S.); octavian.cretu@umft.ro (O.M.C.)

**Keywords:** chronic venous disease, varicose veins, local and systemic oxidative stress, reactive oxygen species, antioxidant systems

## Abstract

Chronic venous disease (CVD) and its major manifestation, varicose veins (VV) of the lower limbs, is a common, multifactorial disease that affects a significant percentage of adult and elderly people worldwide. Its prevalence has been constantly increasing with the aging of the population and, particularly, with the obesity pandemic (hence, the term ‘phlebesity’). The major pathophysiological mechanisms that are potentiating each other in a vicious cycle, leading to chronic venous hypertension, are represented by endothelial dysfunction, chronic inflammation, impaired hemodynamics, and venous wall remodeling. Oxidative stress is another pathomechanism responsible for CVD and its complications, with the increased generation of reactive oxygen species and decreased antioxidant defense being reported to contribute to VV formation. Herein, we present evidence for the role of impaired redox homeostasis as pathophysiological mechanism responsible for chronic local and systemic oxidative stress in patients with CVD.

## 1. Introduction

Chronic venous disease (CVD) of the lower limbs is a common, multifactorial disease that affects a significant percentage of the adult world population and worsens with age [1,2]. CVD has been associated with classic genetical and environmental risk factors, such as family history, female sex, pregnancy (multiparity), occupations with prolonged sitting/standing, and a sedentary lifestyle, yet the most important risk factors remain age and overweight/obesity. The prevalence in the adult population has been estimated at about 77% in women and 57% in men, with the most common form being varicose veins (VV). The most severe form, chronic venous insufficiency (CVI), is more prevalent in women with familial predisposition or in those with a history of multiple pregnancies [3].

Globally, the prevalence is higher in developed countries and, with the aging of the world population and obesity pandemic, it is expected to further increase in the next decades, thus posing a substantial economic burden to healthcare systems [4,5]. Indeed, it was predicted more than one decade ago that over 50% of USA adult population would be obese by 2030 [6], and several cohort studies reported a positive association between obesity and varicose veins (reviewed in ref. [7]) and a negative correlation between the BMI and CVD treatment and outcomes (the higher the BMI, the worse the phlebectomy and/or endovenous therapies outcomes) [8]. Also, there is a clear, albeit less explored link between CVD and diabetes mellitus (DM), another threatening pandemic of this century together with obesity (“diabesity”) [9], since both CVD and DM share the same risk factors [10].

The internationally recognized CEAP (Clinical Etiological Anatomical Pathophysiological) classification of CVP, introduced two decades ago by the American Venous Forum and recently revised [11,12], provides a framework for the description of the venous lesions, the standardization of diagnosis, and decisions about therapeutic strategies [13]. Accordingly, CVD encompasses a broad spectrum of symptoms/signs with increasing severity, from leg discomfort/swelling to venous ulceration in the end stages of the disease [14]; however, varicose veins (VV) remain the most frequent manifestation [15].

VVs are linked to valve insufficiency, venous reflux, and/or venous obstruction, all leading to venous hypertension, increased wall stress, and the activation of venous endothelial and smooth muscle cells [16], which impact microcirculation, ultimately resulting in edema, trophic damage, and venous ulceration [17]. Increased venous hydrostatic pressure, vein obstruction, and dilation in the lower limbs lead to blood stasis, persistent reflux, and altered shear stress [18]. Hemodynamic changes initially promote a hypoxic environment [19] that further triggers the molecular signaling of endothelial dysfunction [20] and structural remodeling of the venous wall [21], leading to disease progression [14]. The hypoxia-driven inflammatory and oxidative changes lead to the venous wall remodeling observed in the VV [19].

Oxidative stress, defined as the imbalance between excessive reactive oxygen species (ROS) generation/release and/or decreased/inefficient antioxidant defense responsible for cellular/molecular damage and/or disrupted redox signaling/control [22], has been extensively studied for past three decades as major contributor to the pathogenesis of most chronic non-communicable diseases (NCDs), including cardiovascular pathologies (reviewed in refs. [23,24,25,26,27,28]).

Oxidative stress has been reported to contribute to VV development and CVD progression towards the chronic venous insufficiency (CVI), both per se and by potentiating the other pathophysiological mechanisms: hemodynamic stress and inflammation [29], the proteolytic activity of matrix metalloproteinases [30], red blood cell release of hemoglobin, DNA damage [31], and venous wall/valvular remodeling [32,33]. These pathomechanisms, which mutually intensify each other in a vicious cycle, lead to the loss of the biophysical properties of the VV walls [34], and in the presence of genetic predisposing factors [20], ultimately result in venous leg ulcers, the most severe complication of end-stage CVI [35].

Several pilot and small cohort human studies have addressed the contribution of oxidative stress to the CVD pathogenesis, with mixed results being reported in the literature due to the heterogeneity of the study design and variety of techniques used for the oxidative stress assessment.

We sought to summarize what is currently known about the role of impaired redox homeostasis in the setting of CVD. Herein, we present evidence for the contribution of increased ROS generation and decreased antioxidant defense as pathophysiological mechanisms, contributing to the stage-dependent local and systemic oxidative stress in patients with CVD; identify the knowledge gaps and potential research directions; and briefly discuss the therapeutic opportunities.

### Data Sources

We searched the PubMed and Google Scholar databases using the following terms (used as single search keywords or in combination): “chronic venous disease”, “chronic venous insufficiency”, “varicose veins”, “varices”, “oxidative stress”, “reactive oxygen species”, “free radicals”, “oxyradicals”, and “enzymatic and non-enzymatic antioxidants”. The search was conducted without date restrictions up to June 2025. We also manually searched the reference lists of all eligible studies (original articles, narrative and systematic reviews, metanalyses) to identify further relevant studies. Studies with measurable outcomes related to oxidative stress, with direct or indirect measurements of either increased ROS generation (and their deleterious consequences) or decreased antioxidant defense in CVD, were included. The review did not address the role of oxidative stress in the venous leg ulcers since it has been comprehensively reviewed elsewhere [36].

## 2. Assessment of ROS Generation, ROS Sources and Markers of Oxidative Stress in CVD

The umbrella term ROS encompasses a group of compounds derived from molecular oxygen, classically divided into free radicals (represented mainly by superoxide, hydroxyl, nitric oxide radical), and non-radical ROS (such as hydrogen peroxide, peroxynitrite, hypochlorous acid). These molecules exert both beneficial effects in human physiology (when generated in small amounts), as signaling molecules responsible for oxidative eustress, and deleterious effects in human pathophysiology (when produced in supraphysiological concentrations), responsible for oxidative distress [37].

Superoxide anion (O_2_•^−^) is the prototype of a free radical generated in multiple cellular compartments of the vascular walls, including the cellular membrane and cytosol, as well as various organelles, such as mitochondria, peroxisomes, and endoplasmic reticulum. The major source of vascular O_2_•^−^ is considered to be the Nicotinamide Adenine Dinucleotide Phosphate/NADPH oxidases (or NOX), in particular NOX1, 2, and 4) [38,39,40], which are multidomain proteins specialized in deliberate ROS production [41]. Vascular NOX isoenzymes are located in all 3 layers—intima (endothelial cells), media (vascular smooth muscle cells), and adventitia (fibroblasts, phagocytes) [42]—and also in the perivascular adipocytes [43]. In the arterial walls, Nox1 and Nox2 promote the development of endothelial dysfunction and inflammation, while Nox4 is considered to have a protective role and becomes detrimental only when its activity increases [43]. NOX enzymes have been reported to be induced by angiotensin II, cytokines, and impaired blood flow in the arterial circulation, stimuli that are also present during CVD progression. At variance from the arteries, the role of NADPH oxidases in the VV has been less explored; the available literature data are presented below.

Superoxide anions are also generated during the increase activity/expression of other enzymes in the vasculature, namely nitric oxide synthases (NOS), in particular the inducible iNOS and uncoupled endothelial eNOS [44], and xanthine oxidase (XO) [45]; their contribution to venous oxidative stress in VV is further presented. Due to its instability and short half-life, superoxide undergoes spontaneous dismutation and is rapidly converted into hydrogen peroxide via superoxide dismutases (SOD) [46].

Paradoxically, the less investigated vascular source of ROS in CVD is the dysfunctional mitochondria, namely the leaking electron transport system (ETS) at the inner mitochondrial membrane. Endothelial mitochondria have emerged as major contributors to the pathogenesis of all chronic diseases associated with vascular injury, where they are both sources and targets of oxidative stress [47,48,49]. As such, in various experimental models, including human umbilical vein endothelial cells (HUVECs), hypoxia and/or reoxygenation increased superoxide production by complex I (CI) and complex III (CIII) of the ETS [50], and/or elicited the reverse electron transport at CI with the same prooxidant effect [51,52]. Furthermore, in hypoxic endothelial cells, ETS-derived ROS were reported to increase NF-kB activation, IL-6 mRNA expression, and endothelial permeability [53], pointing to the pro-inflammatory role of the vascular oxidative stress. Also, HUVECs have been reported to act as mechanosensors since cyclic strain has been reported to double ROS production [54], an observation relevant for VV in the presence of reflux and mechanical stress.

Hydrogen peroxide (H_2_O_2_), the main non-radical ROS, is more stable and acts as the most important signaling molecule when generated in small amounts [55]. However, in pathological settings, besides the superoxide dismutation and NOX4 activity, two other important sources have been reported to constantly generate high amounts of H_2_O_2_ in mitochondria of the vascular walls: monoamine oxidase (MAO), whose activity is increased by chronic inflammation [56] and in the setting of metabolic diseases [57,58], and the growth factor adaptor p66Shc, which modulates both mitochondrial and cytosolic ROS production and contributes to the age- and metabolism-related arterial dysfunction [59,60]. While the role of the latter as an oxidant in the venous wall has not been investigated so far, we have identified MAO as a contributor to the oxidative stress in the VV (Rațiu et al., MS in preparation).

It must be emphasized that both O_2_•^−^ and H_2_O_2_, when overproduced, play deleterious roles, not only as triggers of molecular damage but also as promoters of inflammation, particularly in NLRP3 inflammasome activation [61]. Moreover, in addition to the damage of cellular DNA, proteins, lipids, mitochondria-derived O_2_•^−^ and H_2_O_2_ serve as kindling ROS, which activate NOX isoforms and XO [62] and induce eNOS uncoupling, further amplifying O_2_•^−^ production; the latter, in turn, subsequently inactivates NO, yielding the toxic peroxynitrite (ONOO-), and also decreases eNOS activity and/or expression [63].

At variance from the well-established contribution of ROS in the pathogenesis of arterial dysfunction (reviewed in refs. [44,64,65,66]), fewer studies have been conducted to assess ROS generation, their sources, and deleterious effects in the veins; these studies are further summarized.

### 2.1. Assessment of ROS Generation in CVD

Guzik et al. assessed vascular superoxide anion production by means of lucigenin-enhanced chemiluminescence in proximal and distal segments of varicose veins (VV) harvested from patients undergoing surgery *vs.* the healthy fragments of great saphenous veins (GSVs) obtained from patients subjected to coronary artery bypass graft (CABG) surgery [67]. They demonstrated that VVs generated significantly more O_2_•^−^ as compared to healthy veins, particularly in their distal segments. In healthy veins, superoxide production in the proximal segments significantly correlated with that in the distal segments. NADPH oxidases and uncoupled nitric oxide synthases (NOS) have been identified as sources of superoxide production since their inhibition greatly mitigated the free radical level.

In a pioneering work performed in CVD patients undergoing GSV stripping more than two decades ago, Flore et al. demonstrated a significant reduction in reactive oxygen metabolites (ROMs) generation after the procedure as compared to pre-procedural values [68]. The level of hydroperoxides was significantly higher in the blood collected from the dorsal foot vein of the involved leg prior to GSV stripping as compared to the level measured in healthy controls, regardless of the age and sex of the patients. Since no significant difference in ROS levels harvested from the same site was observed 30 days post-stripping between the groups (with intervention and control), the authors emphasized the beneficial effect of the procedure in mitigating oxidative stress. More recently, a second study from the same group was aimed at investigating the systemic level of various parameters of oxidative stress, including the ROMs in the venous blood drawn from the antecubital vein in CVD patients diagnosed with unilateral (U-CVD) or bilateral (B-CVD) reflux at the saphenofemoral junction as compared with a control group with no venous reflux [69]. In this study, no difference was found in the plasma level of hydroperoxides among the groups. However, a higher level of fibrinogen was found in patients with bilateral reflux, pointing to enhanced inflammatory status in this group that precedes the occurrence of systemic oxidative stress.

Gwozdzinski et al. conducted a pilot study in 8 patients with CVD C2 and no inflammatory pathology, aimed at assessing several biomarkers of oxidative stress in blood samples (plasma, hemolysate and erythrocyte membrane) collected from varicose veins *vs.* the peripheral (antecubital) vein of the same patient [70]. No significant difference was found in the level of peroxides in the membranes of erythrocytes isolated from the VV blood as compared to those prepared from the normal peripheral vein blood, despite the fact that several other markers of oxidative stress were increased along with the decrease in the antioxidant enzymes in the VV (as further described). This observation points to the importance of assessing multiple parameters when investigating the oxidative stress due to the variability in the sensitivity of techniques.

We also recently conducted a pilot study (n = 28) in patients with CVD undergoing cryostripping surgery, a therapeutic procedure suitable for the economic conditions of middle-income countries [71], and reported a significant increase in oxidative stress (superoxide and hydrogen peroxide levels) in varicose veins samples harvested from obese (n = 12) *vs.* non-obese (n = 16) patients, which was alleviated by acute incubation with active vitamin D (calcitriol, 100 nM) [72].

### 2.2. Assessment of ROS Sources in CVD

While Guzik et al. provided pharmacological evidence for NADPH oxidases and uncoupled eNOS as venous enzymatic sources of free radicals (using their pharmacological inhibition) [67], Ortega et al. recently performed an elegant study in the largest group of patients (n = 110) with CVI and assessed the expression of NO synthases (iNOS and eNOS) and two isoforms of the NOX family (NOX1 and NOX2) in great saphenous vein (GSV) samples along with a classic oxidative stress parameter in the serum (the results are described in the next section) [73].

Other enzymes reported to contribute to vascular ROS generation are myeloperoxidase and xanthine oxidase.

Myeloperoxidase (MPO) is a heme-peroxidase secreted by neutrophils and monocytes with an essential microbicidal role via its ability to produce hypochlorous acid (HOCl). High levels of MPO have been reported in cardiovascular diseases and the enzyme has been identified in the atherosclerotic lesions and contributes to the progression of coronary artery disease by oxidizing the LDL particles [74].

Xanthine oxidase (XO) is involved in the oxidation of hypoxanthine to xanthine and of xanthine to uric acid in the presence of xanthine oxidase (XO), resulting in the endothelial generation of both O_2_•^−^ and H_2_O_2_; its expression is increased in patients with coronary artery disease and its inhibition has been reported to alleviate the arterial endothelial dysfunction (recently reviewed in ref. [75]). More than a decade ago, a pioneering study reported the increased activity of XO in the wound fluid collected from patients with chronic venous leg ulcers and postulated that elevated concentrations of XO at the site of chronic wounds may contribute via the local oxidative stress and uric acid generation to wound severity and its delayed healing [76].

At variance from their well-studied role in arteries, only two studies have addressed the contribution of these enzymes in veins.

A pilot study was carried out in 36 patients with indications for VV surgery to assess the contribution (content and localization) of MPO and XO to the local oxidative stress in 3 types of samples, varicose veins (VVs), VVs with superficial thrombophlebitis, and unchanged saphenous veins, as compared to normal veins samples obtained during cadaver organ procurement [77]. The authors reported the highest content of MPO in VVs and slightly lower in the VVs with thrombophlebitis. Immunohistochemical analysis of XO localization showed the positive intense staining of the endothelium in both VVs and VVs with superficial thrombophlebitis; in the latter samples, the enzyme was also present in the vasa vasorum.

Condezo-Hoyos et al. assessed the systemic oxidative stress in a pilot study (9 patients with CEAP C2 stage CVD); they quantified the activities of MPO and XO as ROS generating-enzymes and found no differences in these enzymatic systems in plasma from patients *vs.* controls [78]. They also measured several plasma biomarkers of oxidative damage (i.e., MDA, advanced oxidation products, protein carbonyls, and 3-nitrotyrosine) and those of antioxidant defense (i.e., total antioxidant capacity, total thiols, reduced glutathione, catalase and uric acid). In comparison to the controls, CVD patients demonstrated reduced catalase activity and thiol concentrations, together with elevated MDA-bound protein and protein carbonyls. The values were employed to compute the global index of oxidative stress (OxyVen), which was significantly different *vs.* the control group (10 healthy individuals).

Collectively, these studies showed that the local venous oxidative stress prevailed over the systemic one in patients with CEAP C2 CVD. Of note, a study addressing the arterial oxidative stress reported that oxidative stress at the tissue level (arterial wall) is more accurate as compared to circulating biomarkers for the assessment of prognostic and therapeutic responses in the setting of cardiovascular disease [79]. Whether this holds true with respect to CVD progression is not known.

Table 1 summarizes the clinical studies that assessed ROS levels, as well as their enzymatic sources that have been investigated in the setting of CVD.

**Figure 1:**
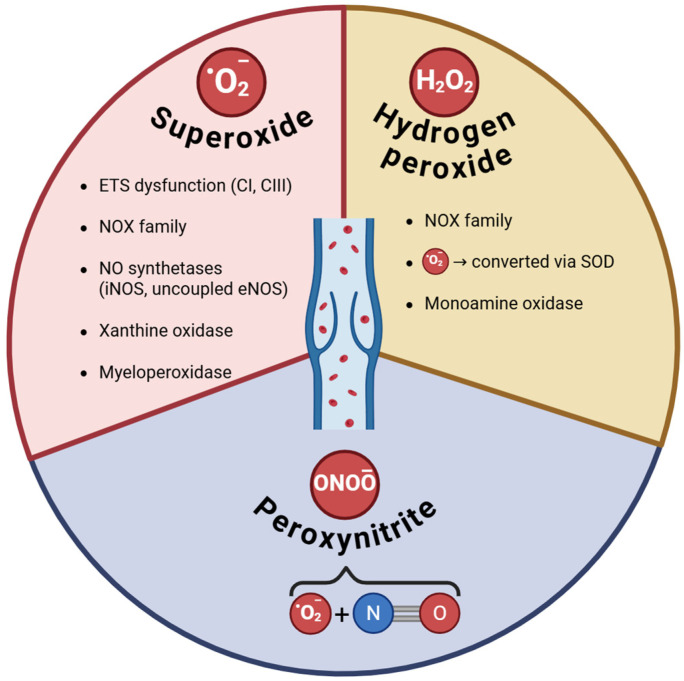
ROS and their sources in varicose veins. ETS—electron transport system; CI, CIII—complexes I and III of ETS; NOX (NOX1 and 2 are the main sources of superoxide, while Nox4 is also a source of hydrogen peroxide); NO—nitric oxide; SOD—superoxide dismutase.

The main ROS along with their sources investigated in CVD are depicted in Figure 1.

### 2.3. Assessment of Oxidative Stress Biomarkers in CVD

#### 2.3.1. Lipid Peroxidation Assays

Lipid peroxidation is a ROS-mediated chain of reactions that results in the oxidative breakdown of polyunsaturated fatty acids. The two major products of the omega-6 fatty acids peroxidation are malondialdehyde (MDA) and 4-hydroxy-2-nonenal (4-HNE), while the F2-isoprostanes are derived from the arachidonic acid; all three are the most frequently measured biomarkers of lipid peroxidation in biological fluids and tissues [80,81].

Malondialdehyde (MDA) is one of the most used biomarkers of lipid peroxidation and also a marker of cell membrane injury. Since its reaction with the thiobarbituric acid evaluates one oxidative stress end-product, nowadays the assay of “thiobarbituric acid reactive substances” (TBARS) is mainly used as a global test for lipoperoxidation [82].

With few exceptions, most studies performed in patients with CVD reported significantly higher levels of MDA/TBARS in the venous plasma.

Pioneering studies were performed by the research group of Stepniewski more than 2 decades ago [83]. As such, in 31 patients (23 females and 8 men) with CVD stages C2 and 3, Krzysciak et al. measured the MDA levels in plasma harvested from the VVs as compared to the antecubital vein from the same patient (as its own control), showing that the local MDA levels in VVs were significantly higher than those in systemic blood in women (but not in men). Furthermore, the diseased group was compared by Kozka et al. with a control consisting of 31 volunteers (20 females and 11 males) with no venous disease [84]. The authors reported significantly increased overall MDA concentration in plasma from all veins in patients with CVD as compared to healthy volunteers. These values were also higher in obese *vs.* normoponderal patients and the body mass index (BMI) substantially correlated with increased plasma MDA concentration. In the same group of patients with CVD C2 and 3, Krzyściak and Kozka measured the levels of tissue MDA in the 3 types of venous fragments harvested from the proximal insufficient GSV, distal sufficient GSV, and VVs (tributaries to GSVs) and reported the highest MDA activity in the VVs and insufficient vein fragments, respectively [85].

Budzyń et al. measured plasma MDA concentration (along with other markers of oxidative stress) in 35 patients (24 women and 11 men) with CVI and reported significantly higher values as compared to a control group of 23 individuals (16 women and 7 men) without signs of CVI [86]. These authors also reported a raised MDA concentration in the female group versus the male group, in line with the findings of Krzyściak et al.; however, it has to be mentioned that women were more numerous as compared to men in both studies [83]. In a more recent study, the same group reported a significant stage-dependent increase in the MDA levels in 44 women with CVI, divided into two severity subgroups—moderate CVI (CEAP C2 and 3) and severe CVI (CEAP C4-6)—versus controls (25 age-matched healthy women) [87]. Similarly, in 9 patients (5 women and 4 men) with early CVD (C2), Condezo-Hoyos et al. reported double the plasma MDA levels compared with healthy controls (5 women and 5 men) [78].

More recently, Palmieri et al. sought to compare the tissue and plasma MDA in venous and arterial dilative pathologies, i.e., in patients with VVs and abdominal aortic aneurysm (AAA) [88]. To this end, they measured the MDA concentration in peripheral venous plasma and tissue homogenates of VVs prepared after varicectomy and AAA samples harvested during the surgical repair of the aneurysm, respectively. The tissue MDA concentration in the VV patients was similar to that in the AAA patients. At variance, the plasma MDA concentration was significantly lower in the VV group versus the AAA group; however, the blood values of MDA in VV patients were higher when compared to the ones in a healthy control group. Interestingly, these authors first reported the occurrence of telomere shortening, detected in endothelial cells from both VV and AAA tissue samples, and postulated that the increased local oxidative stress is responsible for telomere attrition, driving the senescence of the cells in the venous wall.

Saribal et al. also reported increased MDA activity in saphenous vein samples harvested during vein surgery (together with a significant increase in catalase, as a marker of antioxidant defense, in patients with VVs [89]).

Ortega et al. performed the largest study in the field and assessed several oxidative stress parameters in 110 CVI patients, organized according to the presence or absence of valvular incompetence into the reflux group (R) and the non-reflux group (NR), which were further subdivided each in 2 subgroups according to age (50 years = the cutoff value) as follows: NR < 50 (n = 13), NR ≥ 50 (n = 16), R < 50 (n = 32), R ≥ 50 (n = 49) [73]. Patients from the R group exhibited a significant increase in plasma MDA concentration, the indicator of lipid peroxidation, as compared to the NR group (*p* < 0.05). An age-dependent differential distribution of the MDA values was found among the subgroups, with the highest values in R < 50 patients (*p* < 0.005). As previously mentioned, this group also assessed the gene and protein expression of eNOS and iNOS, NOX1 and NOX2. The authors reported the highest percentage for iNOS and eNOS gene expression in patients included in the NR ≥ 50 and R < 50 groups. As for protein expression, NR ≥ 50 patients exhibited greater intensity of iNOS in the adventitia, whereas NR < 50 patients showed the highest intensity in tunica media of the vein walls. Interestingly, eNOS gene expression and protein expression were especially intense in the adventitia of R ≥ 50 patients. mRNA for NOX1 and NOX2 were significantly higher in R < 50 patients *vs.* NR < 50 patients. Interestingly, NOX2 gene expression was higher in the R < 50 group as compared to the R ≥ 50 one. Protein expression for both NOX1 and NOX2 was also the highest in R < 50 patients in all three tunicas of the vein walls. However, NOX1 protein was also overexpressed in the entire venous wall in the NR ≥ 50 patients.

Glowinski measured TBARS in tissue homogenates from varicose veins, unchanged veins, and veins complicated by thrombophlebitis that were harvested during surgery from 36 patients (21 females and 15 males), and compared their level with those of the normal vein homogenates (harvested during cadaver organ procurement) [77]. They reported that TBARS content was the highest in VVs complicated with superficial thrombophlebitis; also, the values in the varicose and unchanged veins were significantly greater than in controls. More recently, Gwozdzinski et al. reported a significantly higher TBARS level in the varicose vein plasma as compared to the peripheral vein plasma from the same CVD patients [70].

Collectively, most of the above-mentioned studies reported high levels of local oxidative stress biomarkers in the VV blood/tissue samples harvested from patients with early CVD, similar to the previous studies assessing local ROS levels.

At variance, few studies reported no differences (or even decreased) MDA/TBARS levels in patients with VVs *vs.* their corresponding controls.

As such, Yasim et al. measured free MDA in the plasma harvested from the brachial vein of 25 patients with primary varicose veins versus 25 healthy volunteers and found no statistically significant difference between the study and control group with respect to the levels of MDA and also of several pro-inflammatory markers (e.g., protein C, fibrinogen, IL-6, homocysteine) [90]. While the authors concluded that the early stages of CVD are not associated with increased systemic oxidative stress, it has to be mentioned that the conjugated form (not the free MDA) is the predominant plasmatic form. Also, the occurrence of high local oxidative stress cannot be excluded (not assessed).

Farbiszewski et al. evaluated the TBARS concentration in varicose saphenous vein fragments with or without thrombophlebitis as compared with the normal veins harvested from the same patient during VV surgery and reported lower levels of TBARS (by 47%) in the VV segments as compared to the normal venous segments [91]. In VVs with thrombophlebitis, the TBARS level was increased by 16% when compared to the normal veins.

#### 2.3.2. Protein Oxidation Assays

Protein carbonylation is the most frequent oxidative modification, irreversibly affecting proteins. The most used method to detect carbonylated proteins consists of protein carbonyl derivatization with 2,4-dinitrophenylhydrazine (DNPH) [92]. Dinitrophenol hydrazone (DNP)-carbonyl can be detected spectrophotometrically or by an immunoassay using specific DNP antibodies. Other sensitive methods for assessing protein carbonyls are ELISA and Western blot [93].

Another strategy to detect oxidative-mediated protein damage is to measure the advanced oxidation protein products (AOPP), an assay used, among others, to assess oxidative stress in the critical ill patients [94] or to predict (in association with imagistic techniques) the occurrence of subclinical atherosclerosis [95].

Nitrotyrosine (3-NT) is another biomarker for protein oxidation. 3-NT is generated as a result of post-translational modification of proteins elicited by the highly reactive peroxynitrite (produced by the reaction between nitric oxide and superoxide anion) and is toxic for the endothelial cells in the setting of cardiovascular diseases [96].

In their pilot study on patients with early CVD (CEAP C2 stage), Condezo-Hoyos et al. reported that plasma protein carbonyls showed approximately double values as compared to controls patients (also for the protein bound MDA mentioned earlier), pointing to the early occurrence of both protein and lipid oxidative damage in the evolution of the disease [78]. However, they also measured the plasma levels of AOPP and total 3-NT, and found no statistically significant differences between the groups.

Of note, increased nitrotyrosine, as an indicator of the presence of ONOO-, has been reported to occur in wound biopsies from both acute and chronic venous leg ulcers (in association with protein carbonylation and lipid peroxidation) [97]. In fluids from chronic (but not acute) wounds, radical scavenging activity and glutathione level were elevated. Since oxidative stress also potentiates local inflammation, the authors speculated that an increase in antioxidant defense in the advanced CVI occurs as a compensatory mechanism against chronic inflammation.

Gwozdzinski et al. examined the protein carbonyl concentration in the plasma and erythrocyte membrane of VV patients [70]. They reported higher levels in VV plasma as compared to peripheral vein plasma. Similar results were observed when analyzing protein carbonyl compounds in the erythrocyte membrane, which were also higher in expression in the VV blood than in the peripheral vein blood.

#### 2.3.3. Prolidase Enzyme Activity

Prolidase is a ubiquitously expressed cytosolic metalloproteinase with crucial roles in collagen turnover and matrix remodeling in conditions associated with inflammation and cell proliferation (recently reviewed in ref. [98]); it is also considered a marker of oxidative stress since its activity has been reported to increase in association with other oxidative stress biomarkers in both acute and chronic pathologies [99,100,101].

Akar et al. evaluated the global oxidative status (the total oxidant status—TOS; the oxidative stress index—OSI)) and the prolidase enzyme activity in varicose veins and serum of CVD patients subjected to superficial vein surgery (n = 30) and used two control groups, a serum control group (healthy blood from patients without CVI, n = 30) and a tissue control group (healthy fragments of the great saphenous veins from patients undergoing coronary artery bypass graft surgery, n = 30), respectively [102]. These authors demonstrated that both local oxidative stress and prolidase enzyme activity were higher in the tissue fragments from the VV group as compared to the tissue control group. At variance, neither the oxidative stress parameters (TOS and OSI) nor plasma prolidase activity were significantly different in the serum samples from the VV patients and the corresponding controls. The findings strongly suggest the major contribution of the local redox dyshomeostasis and collagen impairment to the venous wall remodeling.

Table 2 summarizes the clinical studies that assessed the biomarkers and deleterious consequences of oxidative stress in CVD.

## 3. Assessment of the Antioxidant Defense in CVD

The constant generation of ROS in the human body is regularly inactivated by a complex battery of endogenous and exogenous antioxidants; this battery maintains redox homeostasis, prevents cellular damage in healthy tissues, and is progressively depleted during disease [105,106]. According to Halliwell and Gutteridge, there are 3 types of antioxidants: (i) primary antioxidants, which prevent the formation of oxidants, (ii) secondary antioxidants, which act as ROS scavengers, and (iii) tertiary antioxidants, which facilitate the repair of oxidized molecules [107].

A lower concentration of antioxidants signals the exhaustion of one or more lines of antioxidant defense and plays a significant role in oxidative stress development.

Total antioxidant capacity (TAC) is a widely used metric to quantify the ROS-buffering capacity of biological samples, indirectly providing information about the magnitude of oxidative stress [108]. The currently used assays measure TAC directly (i.e., assess the ability of a sample to inhibit the oxidation of a substrate) or indirectly (i.e., assess the ability of a sample to reduce a metal, usually Fe^3+^ and Cu^2+^). The former groups of tests include the total radical trapping antioxidant parameter (TRAP), oxygen radical absorbance capacity (ORAC), and Trolox equivalent antioxidant capacity (TEAC), while to the latter group belong the ferric-reducing ability of plasma (FRAP) and the cupric-reducing antioxidant capacity (CUPRAC) assays.

Budzyn et al. monitored TAC in plasma obtained from CVI patients and a control group. TAC was significantly higher in CVI patients compared to the control group, with the highest values recorded in patients assigned to the subgroup S (with severe symptoms) and group II (with more than 10 years of disease duration), respectively [86]. These authors reported that CVI women had higher TAC values than females in the control group. When performing the sex analysis, significantly lower TAC levels were found in CVI women *vs.* CVI men. Moreover, TAC was influenced by the weight of CVI patients, with patients with a BMI > 25 having higher TAC levels than those with a BMI < 25. Of note, in this study, TAC was not influenced by age.

Horecka et al. measured TAC (and other antioxidants) in both tissue and blood samples from VV patients CEAP C2 CVD patients (n = 65) *vs.* two control groups, with one for tissue—control group 1 (normal great saphenous vein samples collected from 10 patients subjected to CABG surgery)—and the other for plasma—control group 2 (blood samples collected from 20 healthy individuals) [109]. Both tissue and plasma TAC were significantly decreased in patients as compared to the corresponding control group. The authors concluded that there was an impaired antioxidant defense in the blood of these CVD patients; however, the fact that the early stage of the disease may not have resulted in a major activation of the antioxidant defense that could be reflected by the TAC assay cannot be excluded.

Akar et al. evaluated TAC in both tissue and serum samples and reported a significant decrease in tissue TAC level and no differences in circulating TAC in VV patients *vs.* controls [102]. Similarly, no significant differences regarding plasma TAC levels were found by Condezo-Hoyos et al. in their study on CVI patients undergoing vein surgery *vs.* controls [78].

FRAP, often used as a measure of total antioxidant power (TAP), assesses the ability of antioxidants to reduce ferric ions (Fe^3+^) to ferrous ions (Fe^2+^). Krzyściak et al. conducted a study in which they found significantly lower FRAP values in VV blood compared to peripheral (antecubital) vein blood from the same patient, thus indicating a decreased capacity for ROS scavenging in the diseased vessels. Decreased FRAP levels were also observed in the blood harvested from the VVs as compared to the antecubital vein blood in CVI women; interestingly, no such differences between the two blood specimens were found in men with CVI [83]. In the same group, TAP was measured in tissue samples from insufficient proximal GSVs, sufficient distal GSVs, and varicose saphenous tributaries to GSVs. They found that the values were lower in VV and insufficient vein fragments *vs.* the sufficient vein segments [85].

In a pilot group of VV patients, Condezo-Hoyos et al. measured TAC and ORAC as biomarkers of the plasma antioxidant systems and found no differences *vs.* controls [78]. These authors also assessed other non-enzymatic antioxidants. While uric acid (UA) levels were similar in the two groups, total plasma thiols were significantly decreased in the patients and GSH showed a decreasing tendency, albeit not reaching statistical significance due to the reduced number of patients.

Gwozdzinski et al. conducted a study that evaluated another global antioxidant biomarker, i.e., non-enzymatic antioxidant capacity (NEAC) [70]. They investigated alterations in plasma and red blood cell properties in patients with VVs. Plasmatic NEAC was analyzed using two independent methods and was found to be decreased in VVs as compared to peripheral veins from the same subjects in both cases.

Table 3 summarizes the main clinical studies that globally assessed the antioxidant capacity in patients with CVD.

### 3.1. Assessment of Enzymatic and Non-Enzymatic Antioxidants in the Setting of CVD

In order to mitigate the detrimental effects of oxidative stress, the human body has developed a sophisticated, three-line antioxidant defense system that operates in concert and is effective in maintaining healthy levels of intracellular oxyradicals within physiological limits [105]. Primary antioxidants inhibit oxidant formation; secondary antioxidants function as scavengers of ROS, and tertiary antioxidants repair the oxidized molecules.

The first line and most powerful enzymatic antioxidant defense system consists of several enzymes, the most important being: (i) superoxide dismutases (SOD), (ii) catalase (CAT), (iii) glutathione peroxidase (GPx), (iv) thioredoxins (Trx), and (v) peroxiredoxin (Prx) [110].

The second line of defense is represented by the non-enzymatic antioxidants able to rapidly inactivate oxidants/free radicals, which include: (i) thiols, the most abundant intracellular compound being glutathione (GSH) [111], (ii) vitamins (A, C, E), (iii) uric acid, (iv) bilirubin, (v) carotenoids, and (vi) polyphenols [112].

The third line of defense comprises enzymatic antioxidants that counteract the ROS-induced damage by repairing damaged DNA and proteins, removing oxidized molecules (e.g., lipids), and restoring deteriorated cellular membranes and components [105,112].

#### 3.1.1. The Role of Enzymatic Antioxidants in CVD

The most commonly investigated enzymatic antioxidants in the setting of CVD were superoxide dismutase, catalase, and glutathione peroxidase.

(a) **Superoxide dismutase (SOD)**

SOD is the most powerful cellular antioxidant that directly reacts with an oxyradical; namely, it catalyzes the dismutation of superoxide radical into hydrogen peroxide and molecular oxygen. In mammals, there are three isoforms of SOD: SOD1 (homodimeric Cu/ZnSOD), primarily found in the cytoplasm and nucleus of cells and the intermembrane mitochondrial space, SOD2 (MnSOD), located in the mitochondrial matrix and SOD3 (tetrameric Cu/ZnSOD), the extracellular form [105].

SOD was the most frequently assessed enzyme in the setting of CVD and mixed results have been reported in the literature.

Wali et al. performed a pioneering pilot study (24 patients with CVD undergoing vein surgery) and harvested vein specimens from both the stripped mid-thigh great saphenous vein (GSV) and the distal calf varicosities (a total of 44 samples) [113]. They reported an 80% higher SOD activity in the distal calf varicosities than in the mid-thigh GSV and a mean concentration of superoxide (the SOD substrate) in the wall of the distal calf varicosities that was twice as high as the one from the mid-thigh GSV samples.

Krzyściak et al. conducted a study in 31 patients with CVD CEAP C2/3. During surgery, they harvested 3 types of venous segments from proximal insufficient GSVs, sufficient distal GSVs, and varicose GSV tributaries [104]. They assessed the local antioxidative status by measuring the tissue SOD and glutathione peroxidase (GPx) activities and reported a significant increase in both enzymes in the insufficient and varicose veins as compared to the sufficient ones. They also measured the iron content in venous vessels by means of Proton-Induced X-ray Emission Spectroscopy (PIXE) and also analyzed the DNA oxidative damage by the Comet method. They reported an increased iron deposition in the incompetent and varicose veins (as compared to controls) that resulted in DNA damage. These authors also reported DNA damage in the peripheral blood lymphocytes isolated from the patients with CVD [114]. Of note, the same group also measured the concentration of copper and zinc ions in lyophilized sufficient and insufficient GSVs and VVs and reported high values that correlated with the increased SOD activity in the venous homogenates prepared from the diseased veins [115].

Horecka et al. conducted a study in CEAP C2 CVD patients (n = 65) and reported decreased total antioxidant status in both peripheral blood and varicose veins. These authors also measured SOD activity in the VV and erythrocytes from the peripheral blood collected from the antecubital vein during surgery [109]. They reported a significant decrease in the activity of SOD in erythrocytes of patients with VV but enzymatic overactivity in the venous walls, presumably as a compensatory mechanism. The reduced activity of SOD and catalase in the plasma of patients with VVs as compared to controls was also reported by a study carried out more than two decades ago [116]. At variance with this, Farbiszewski et al. reported that SOD activity was significantly decreased (not increased) in the segments of saphenous vein varices as compared to the normal segments. Interestingly, these authors also found that SOD activity remained nearly unchanged in the VV fragments with thrombophlebitis [91].

The above-mentioned results in plasma and VVs were recapitulated by two more recent studies. Karamalakova et al. assessed both enzymatic (SOD, CAT, GPx) and non-enzymatic (reduced glutathione/GSH) antioxidant defense systems in patients with CVI CEAP C_2-4_ (n = 37) and in CEAP_2_ CVI patients with type 2 diabetes mellitus (n = 5) as compared to healthy controls (n = 25) [103]. They reported a significant decrease in plasma SOD activity in the CVI groups compared to controls; additionally, SOD activity was significantly lower in the CVI + DM2 group than in CVI patients. More recently, Modaghegh et al. demonstrated in a pilot study (n = 10) that SOD levels (and MDA) were lower in tissue fragments of the varicose GSVs compared to the non-varicose veins [117].

There is also one null study regarding SOD in the literature published by Saribal et al. [89]. These authors measured the SOD levels (together with CAT, GPx, and glutathione S-transferase/GST) in saphenous veins samples harvested from patients (n = 52) with varicose veins and compared with healthy veins obtained from patients undergoing CABG (n = 52). They found no significant differences between the groups for the tissue levels of SOD, GPx and GST, except for CAT, the level of which was increased. As a limitation of this study, the plasma level of these antioxidants was not assessed.

(b) **Catalase (CAT)**

Catalase (CAT), a heme-containing protein, belongs together with glutathione peroxidase (GPx) to the first-line antioxidant defense, being responsible for the removal of hydrogen peroxide (H_2_O_2_). Specifically, CAT is a classic H_2_O_2_ scavenger with an extremely high turnover that decomposes hydrogen peroxide into molecular oxygen and water, protecting the cell membrane and organelles against peroxidation.

Saribal et al. assessed tissue CAT activity in venous samples obtained during VV surgery and CABG surgery (controls) and reported a significant increase in activity in VV patients [89]. In contrast, Condezo-Hoyos et al. and Karamalakova et al. compared plasma CAT activity in CVD patients *vs.* healthy controls and found significantly decreased activity in CVD [78]. Gwozdzinski et al. also observed lower CAT activity when comparing VV hemolysate and peripheral vein hemolysate from the same subjects with CVD [70].

(c) **Glutathione peroxidase (GPx)**

Glutathione peroxidase (GPx) is a family comprising eight distinct isoforms of GPx (GPx1–GPx8), with GPx1 being the most abundant in humans.

Specifically, GPx utilizes reduced glutathione (GSH) as a substrate for the H_2_O_2_ dismutation to H_2_O, thus detoxifying different hydroperoxides (including lipid hydroperoxides). The enzyme glutathione reductase (GR) then catalyzes the regeneration of reduced glutathione (GSH) from its oxidized form (GSSG), utilizing NADPH [105].

The only study showing a positive modification is that of Krzyściak et al., which reported a significantly increased GPx activity in insufficient and VV fragments compared to sufficient segments of distal GSVs harvested from the same subjects during VV surgery [104]. Conversely, Karamalakova et al. showed the opposite trend when analyzing plasmatic GPx activity that significantly decreased in CVI and CVI + DM2 patients compared to healthy controls, as well as in CVI + DM2 compared to CVI patients [103].

Farbiszewski et al. studied glutathione reductase (GR) activity in 3 types of tissue harvested during VV surgery, VVs, thrombosed vein segments, and normal GSV segments, and found no significant differences among the groups [91].

Glutathione S-transferases (GSTs) are a group of enzymes essential for cellular detoxification, catalyzing the GSH conjugation to various deleterious chemicals [118]. Saribal et al., comparing GPx and GST activity in VV tissue and healthy GSV harvested during CABG surgery, found no statistical difference between the two groups [89].

Literature data regarding the several enzymatic antioxidants in CVD are summarized in Table 4.

#### 3.1.2. The Role of Non-Enzymatic Antioxidants in CVD

A number of studies assessed the non-enzymatic antioxidants in biological samples (tissue or plasma), such as glutathione (GSH), thiols, uric acid (UA), and vitamins (A, C, E) from CVD patients.

(a) **Glutathione (GSH)**

GSH, the most abundant non-enzymatic endogenous antioxidant in the human body (intracellular concentrations of 1–10 mM), is a low-molecular-weight thiol that directly neutralizes harmful reactive oxygen and nitrogen species. Glutathione exists in two free forms: the reduced (GSH) thiol and the oxidized (GSSG) disulphide forms; in physiological settings, almost 98% of total glutathione exists in the reduced form. The molecule is essential for maintaining cellular redox equilibrium and regulating the cell cycle/regeneration, apoptosis, and immune defense [105,112]. It restores other oxidized small molecule antioxidants, such as vitamin C and vitamin E, participates in the repair of protein molecules, nucleic acids, and lipids damaged during peroxidation processes, and maintains the reduced state of protein sulfhydryl groups.

The contribution of GSH to the pathogenesis of arterial endothelial dysfunction and atherosclerosis has been largely investigated over the past decades in relation to nitric oxide (NO) changes. The NO effects have been classically associated with the guanylyl cyclase activation and the subsequent generation of cyclic GMP. Prasad et al. conducted a pilot study in patients with ATS or risk factors (n = 17) and assessed the endothelium-dependent relaxation with acetylcholine (ACh) and endothelium-independent relaxation with nitroglycerin and sodium nitroprusside, respectively, prior to and after GSH administration in the femoral vein [119]. The authors reported that GSH alleviated the endothelial-dependent vasorelaxation by increasing the NO bioavailability (demonstrated by te elevation of cGMP levels in the femoral vein during ACh infusion) and had no influence on endothelium-independent vasorelaxation with either NO donor. However, nowadays it has become evident that the main NO actions are modulated via the posttranslational modification of protein function (by reacting with cysteine residues) referred to as S-nitrosylation and the list of these modified proteins is still expanding [120]. By far less information is available regarding the GSH level in the venous system. Pioneering work performed two decades ago by Aucoin et al. in bovine coronary venular endothelial cells exposed to oxidative stress demonstrated a reduction in the intracellular GSH levels [121].

There are few studies in the literature that investigate the antioxidant role of GSH in humans. Condezo-Hoyos et al. found a slight reduction in plasma level of GSH in VV patients compared to controls, but it did not reach the level of statistical significance [78]. Horecka et al. assessed GSH levels in the varicose vein wall and venous plasma from the antecubital vein of 65 patients with CVD CEAP2 as compared to normal great saphenous vein walls collected from patients who underwent coronary artery bypass (CABG) graft and blood collected from 20 healthy individuals and found no significant difference [109].

Karamalakova et al. assessed the plasma GSH level in CVI patients (CEAP stages C2- C4) and patients with CVI + type 2 DM *vs.* healthy volunteers (controls) [103]. They reported a significant decrease in CVI + type 2 DM (but not in CVI) as compared to controls (*p* < 0.05), indicating higher oxidative stress in the presence of metabolic disease. This requires increased antioxidant defense systems that will exhaust over time.

More recently, Gwozdzinski et al. conducted a small pilot study in 8 patients with VVs and measured the GSH concentration in both plasma and hemolysate (erythrocytes) prepared from the venous for each patients collected from 2 different sites: the antecubital vein before surgery and the VVs during surgery [70]. No statistical difference was noticed between either type of sample.

(b) **Thiols**

Endogenous thiols, compounds that contain a carbon-bonded sulfhydryl group (-SH), participate in both redox signaling and the regulation of various biological processes, as well as in plasma and tissue antioxidant defense [122,123]. Mixed data are available in the literature with respect to the total thiols in patients with CVD.

In an early pilot study, Condezo-Hoyos et al. investigated whether oxidative stress, constantly reported to occur in the VV, is also reflected in the plasma [78]. They assessed, among several other parameters, the level of plasma thiols in a group of CEAP C2 CVD patients and reported decreased plasma thiol levels in CVD patients *vs.* controls.

More recently, Gwozdzinski et al. assessed various biomarkers of oxidative stress in both plasma and red blood cells obtained from varicose veins *vs.* peripheral veins [70]. Specifically, they assessed the properties of plasma, erythrocyte membranes, and hemolysate obtained from the varicose vein blood *vs.* those of the normal peripheral vein blood (used as controls) collected from the same patient. They reported a decrease in total thiols in the erythrocyte hemolysate and membranes, as well as in plasma from the varicose vein blood, in comparison to the corresponding values from the peripheral veins (controls). Since the lower thiols were associated with decreased CAT activity in the VVs, the authors postulated that high local oxidative stress is responsible for the inefficiency of the antioxidant system. Of note, a comparative study of varicose *vs.* systemic blood samples was conducted by Poredos et al. in CEAP C2 patients with respect to the inflammatory and pro-coagulant markers [124]. In blood samples harvested from the leg VV, these authors reported increased values of highly sensitive C-reactive protein, IL-6 and von Willebrand factor, as well as of D-dimers, demonstrating the presence of a pro-inflammatory and pro-coagulant local environment.

At variance from the above mentioned results obtained in blood samples, Modaghegh et al. compared tissue fragments of varicose veins *vs.* control samples (healthy veins used for CABG or harvested from trauma patients) and reported significantly increased total thiols in VV samples compared to both types of controls, suggesting an increased local tissue antioxidant defense [117].

It must be mentioned that in cells, thiols are present in high concentrations (and in reduced state), whereas in plasma, they are found in lower concentrations (and mostly oxidized) [125]. The major plasma thiol that may contribute to the antioxidant defense in the extracellular space is albumin, a versatile molecule with various ligand-binding properties, multiple enzymatic activities (e.g., paraoxonase, thioesterase, glutathione peroxidase, etc.), and free radical-scavenging capacity [126]. Its role in the setting of CVD is worth further investigation.

(c) **Uric acid (UA)**

Uric acid is the end-product of purine metabolism, which has been extensively studied for its prooxidant effects in the setting of cardiometabolic and renal diseases.

Nowadays, its dual nature has been acknowledged, displaying antioxidant properties in a hydrophilic environment, such as plasma and a prooxidant action within the cells [127].

Soluble UA is a crucial antioxidant compound, contributing to approximately 55% of the extracellular capacity for free radical neutralization, and it has been postulated by some authors that the elevation of circulating uric acid levels may serve as an adaptive mechanism to mitigate the harmful consequences of oxidative stress but the underlying pathomechanisms are far from being unveiled. A recent study investigated whether increased circulating UA is an adaptive protective response against the increased ROS levels present in obese individuals. The authors reported that the antioxidant capacity, assessed through FRAP scavenging and CAT activity, was markedly elevated in the obese group relative to the normal weight group and the levels of UA positively correlated with FRAP and CAT activity in individuals classified as overweight and obese [128]. UA-mediated neuroprotection against oxidative stress and neuroinflammation in different stages of neurodegenerative diseases has been ascribed to the activation of the Akt/GSK3β signaling pathway [129].

As regarding the vessels, early studies focused on the role of UA as a prooxidant. Michiels et al. firstly hypothesized that hypoxia impact on endothelial layer initiates a sequential process, originating from reduced oxygen availability, which involves various cell types as a primary cause of venous disorders, besides the genetic and mechanical factors [130]. The low ATP availability during oxygen deprivation of the human venous endothelial cells from umbilical cords (HUVECs) results in calcium-dependent activation of the endothelial cells, synthesis of pro-inflammatory molecules (such as platelet-activating factor), and adhesion of polymorphonuclear neutrophils to HUVECs. Pioneering experimental studies on HUVECs also showed that hypoxia activated xanthine oxidase, resulting in oxidative stress [131]. The conversion of hypoxanthine into xanthine and, subsequently of xanthine into uric acid by xanthine dehydrogenase, results in the generation of two superoxide anion radicals. When administered in healthy volunteers, UA elicited a significant increase in serum free-radical scavenging capacity that was greater than the one induced by the same dose of vitamin C [132].

As for the venous pathologies, Budzyń et al. measured several markers of oxidative stress in 35 CVI patients (n = 35) as compared to a control group (n = 23 individuals with no signs of CVI) [86]. CVI patients were further divided into different subgroups based on the duration and the clinical severity of the disease. The duration groups included group I (<10 years) and group II (>10 years), while the clinical severity groups included group M (mild clinical symptoms of CVI, CEAP C2 or C3) and group S (severe clinical symptoms of CVI, one of C4, C5 or C6 stages). Aspects related to sex, age and BMI influence were also assessed. These authors found significantly lower UA levels in the group with mild clinical symptoms and in the group of CVD patients with less than 10 years of disease progression, compared to the healthy controls. Moreover, they reported significantly lower plasma UA in CVD women as compared to CVD men; the decrease persisted when CVI women were compared with the female controls (but not for CVI men). Conversely, Condezo-Hoyos et al. reported no difference in UA level in VV patients *vs.* controls [78].

(d) **Ascorbic acid**

Ascorbic acid is known as one of the basic exogenous vitamins with tremendous antioxidant characteristics. It is associated with small molecule antioxidants like GSH and tocopherol but also stimulates the activation of antioxidant enzymes like SOD, CAT, or GPx [133]. Farbiszewski et al. demonstrated that ascorbic acid from VV segments was significantly decreased compared to normal GSV segments [91]. At variance with this, significantly higher ascorbic acid levels were reported in VVs associated with superficial thrombophlebitis as compared to normal GSV segments.

The changes in non-enzymatic antioxidants reported to occur in CVD are summarized in Table 5.

Despite unequivocally demonstrating the role of oxidative stress in the pathogenesis of CVD (excellently reviewed in ref. [32], with an emphasis on the contribution of red blood cells to the varicose vein pathophysiology), numerous discrepancies and inconsistencies have been reported among studies tackling local oxidative stress in varicose veins and systemic ones in peripheral venous blood, largely attributable to the different methodologies employed to address research questions, as well as the characteristics of the study groups (including patients’ sex, disease stage, comorbidities and, also, the heterogeneity of the control ones).

This necessitates the execution of larger mechanistic studies to elucidate the contribution of ROS sources implicated in the CEAP stages of CVD with and without comorbidities/risk factors and ultimately, delineate biomarkers that can be targeted and potentially utilized in the formulation of innovative targeted antioxidant and anti-inflammatory therapies.

It has to be mentioned that several position papers and critical reviews have been published in the past decade mentioning challenges and limitations [134], pitfalls in ROS assessment in biological samples [135], the unmet need for a greater precision in their measurement [136], potential errors in performing the assays [137], as well as the need to interpretate the results in the clinical context [108], aspects that also hold true for the above-summarized studies in patients with CVD.

## 4. Discussion

We have presented herein an overview of the studies tackling the contribution of local (in the VV) and systemic (in the peripheral venous blood) oxidative stress to the pathophysiology of CVD. In the first section, we addressed the role of oxidants and summarized the studies that have measured ROS levels and their sources in patients with CVD, whereas in the second section, numerous studies covering the role of various antioxidant systems were presented.

While oxidative stress unequivocally contributes to the pathophysiology of CVD, several issues remain to be elucidated since all the studies published so far were small and non-randomized. Most of these studies reported local increases in various ROS and/or some of their enzymatic sources in the VVs, while mixed results are available in the literature with respect to the systemic oxidative stress. As presented in the summative Table 2, Table 3, Table 4 and Table 5 regarding the biomarkers of oxidative stress and of enzymatic/non-enzymatic antioxidant defense, data reported in the literature for the systemic oxidative stress in the peripheral venous blood showed either increased or decreased values. In the case of the latter, the authors speculated either an incipient disease that did not result in an increase in the oxidative stress biomarkers or an advanced stage of CVD where the decreased values of the antioxidants pinpoints to the exhaustion of the defense systems.

Importantly, the fact that classic oxidative stress (increased prooxidants and/or decreased antioxidants) is a pathophysiological mechanism in the vasculature of patients with advanced cardiometabolic diseases was reported back in the 2000s [138]. However, it has been emphasized already by that time that in the early stages of these diseases, alterations predominantly occur within individual cellular compartments or specific enzymes, without affecting the overall (systemic) redox status. eNOS uncoupling, which results in the production of superoxide (instead of NO) in the cytosol, or superoxide leakage from the dysfunctional ETS in the mitochondria were the most studied local changes that disrupted redox signaling. Moreover, a word of caution has been formulated more than a decade ago with respect to the antioxidant therapies [139], which eventually also hold true for an ROS increase in varicose veins. While sustained elevated ROS levels lead to cellular damage, moderate increases in intracellular oxidants that function as signaling molecules will activate host defense pathways/adaptation to stress, in line with the hormesis concept [140], ultimately leading to a reduction in ROS. This also explains why therapeutic interventions targeting total cellular redox status, such as antioxidant vitamins, were inadequate to alleviate vascular oxidative stress. These findings boosted the research on pharmacological approaches aimed at restoring the function of specific enzymes implicated in ROS generation. Furthermore, they may be equally relevant for venous pathologies.

The enzymatic sources of local venous oxidative stress in the VVs were scarcely addressed in the literature.

NADPH oxidases are a family of membrane oxidoreductases that has been widely acknowledged as a ‘professional’ source of ROS (mainly, superoxide anion) in cardiovascular pathologies [141] underlying the oxidative stress-mediated endothelial dysfunction, both in systemic circulation and microvascular beds [142,143,144]. Of note, their contribution to microvascular endothelial dysfunction has also been reported in sedentary obese young individuals [145], an observation relevant for the CVD where obesity is the most frequent comorbidity.

Also, the contribution of the individual NOX isoforms to the oxidative stress in human varicose veins was inadequately examined. Elegant experiments performed by Guzik et al. more than two decades ago in human saphenous vein samples harvested from patients undergoing CABG shed light on the crucial role of the NOX family in venous oxidative stress. These authors firstly reported that saphenous veins from patients with ATS generate superoxide mainly via an NADPH-dependent oxidase and this was associated with a decrease in NO-mediated vasorelaxation [146]. The role of the NOX family in the early stages CEAP C0-C2 of CVD requires thorough investigation in order to provide early pharmacological therapies.

Another important issue is related to the crosstalk and potentiation of various ROS sources. As such, vascular NADPH oxidases were found to be crucial in the regulation of superoxide production by uncoupling eNOS and XO in the arterial role [147]; this direction is worth being exploited in CVD in order to provide the rationale for combined therapies.

Similar to the NOX family, the contribution of mitochondria impairments to the arterial dysfunction has been largely addressed in the past decades in relation to cardiometabolic pathologies and the cardiovascular risk [148,149]. The regenerative vicious cycle of ROS formation in mitochondria, denominated “ROS-induced ROS release” [150], provides the integration of redox signaling but also the amplification of regional ROS production including in the vascular arterial bed [151]. This process has not been investigated so far in venous circulation.

While vascular ROS has been reported to mediate the plethora of signaling pathways that underlie endothelial dysfunction, inflammation, and impaired lipid metabolism in atherosclerosis and cardiometabolic diseases [152,153], investigations of the contribution of abnormal ROS signaling to the impaired intercellular communication and stress adaptation have started [154]. A similar approach might be useful in the early stages of CVD where no systemic increase in a specific ROS is evident.

Cell-to-cell redox communication entails direct interactions, such as gap junctions where the transfer of chemicals and secretion of redox-active molecules or several types of extracellular vesicles into the extracellular milieu convey these signals to proximate or remote target cells [155]. The study of these aspects in the venous bed is in its infancy.

Since chronic inflammation and oxidative stress are closely intertwined processes, which can promote each other in toxic positive feedback that damages the arterial walls [156,157]; a similar cooperative and synergistic partnership occurs in the venous pathologies. It has been postulated that oxidative stress triggers general inflammatory signaling [158], which further amplifies, according to a positive feed-back loop, the oxidative stress via excessive ROS production and/or the depletion of antioxidants in the setting of NCD [159]. It has been suggested that assessing oxidative stress and inflammation in the initial phases of NCD may serve as an effective tool for prompt intervention and prevention.

Oxidative stress is also tightly linked with chronic inflammation in the evolution of most NCD [160] and also of malignancy [161]. Thus, it has been proposed that the quantification of both redox and inflammatory status is required for the appropriate assessment of a particular pathological condition and for an adequate therapeutic approach. The interdependence of these two pathomechanisms concomitantly occurring in all cardiometabolic pathologies might also explain the failure of most antioxidant trials who did not target both of them [162]. Moreover, monitoring markers of both oxidative and inflammatory status may facilitate earlier and more effective strategies for preventing NCD and/or and assessing their treatment efficacy [159]. However, the inflammatory status was scarcely assessed in the studies that investigated venous oxidative stress. We believe that assessments of both oxidative and inflammatory activity markers should be performed in the early stages of CVD to enable the effective prevention of the disease progression and also therapeutic monitoring.

The vicious circle between oxidative stress and inflammation has been reported to occur in the diseased arteries and also in adipose tissue [163]. Since chronic inflammation has been correlated with the increased severity and progression of the CVD in the setting of obesity [164], investigations of the magnitude of oxidative stress in both venous walls and adipose tissue in patients undergoing elective surgery could also be designed.

Moreover, aging has also been postulated to accelerate the “vascular health triad”, namely, increased oxidative stress, sterile inflammation, and endothelial dysfunction, thus leading to cardiovascular and metabolic complications [165]; studies addressing age-related local and systemic oxidative stress in cohorts of CVD patients are also needed.

A comprehensive investigation of ROS sources, metabolism, signaling, and modulatory effects in the diseased veins should establish a robust foundation to understand their role in the pathogenesis of one of the most frequent chronic diseases, potentially facilitating advanced applications in its diagnosis, prevention, and personalized approach. The main ROS, superoxide anion and hydrogen peroxide, are important signaling molecules in the venous walls where they can trigger numerous signaling pathways, e.g., PI3K/Akt/mTOR [166] and the expression of transcription factors, which may further set in motion the antioxidant defense mechanisms [167].

The abnormal expression of mRNA, protein levels, and proteolytic activity of matrix metalloproteinases (MMPs) has been reported to systematically occur in VVs [168]. However, mechanistic approaches aimed at elucidating the multiple molecular and mechanosensitive signaling pathways in the venous walls in multicentric, randomized trials are mandatory. One direction might be the assessment of GDF15, a well-established marker of arterial oxidative stress, inflammation, and cellular aging in cardiometabolic diseases [169] during the endothelial-to-mesenchymal transition, which is a phenomenon elicited by shear stress in human varicose veins that contributes to their fibrotic remodeling [170,171]. In some CVD patients, this is mediated by TGF-β [172]. Recently, bioinformatic analysis and machine learning have been used in order to explore the potential molecular mechanisms/signaling pathways [173] and genetic risk factors involved in the VV development [174].

Moreover, identifying the major ROS targets in the diseased venous walls (receptors, carriers, enzymes channels) will prove causality with the loss of cellular function, ultimately providing molecular targeted therapies. Also, an appropriate understanding of the roles of the antioxidant enzymes (e.g., glutathione peroxidases and peroxiredoxins), acting as potential sensors of hydroperoxides, and the consequences of the subsequent signal transduction in the setting of CVD represent other less explored areas. The redox networks present in the microcompartments at subcellular levels, in cells, and in tissues are highly dynamic systems adapted to maintain health in a changing environment for a long period of time; when functionally impaired, the redox imbalance also leads to organ failure and disease [175] in the venous circulation, pointing to the importance of the dynamic assessment of the oxidant–antioxidant biomarkers within the CVD progression.

Oxidative stress is druggable and has been systematically investigated in the context of cardiovascular diseases, with both pharmaceuticals, such as statins, a couple of beta-blockers, and RAAS inhibitors [176], and natural antioxidants being administered for the vascular health [177]. As for CVD, the current therapeutic management of VV comprises conservative approaches (leg elevation, compressive stockings), pharmacological treatment (venotonic drugs and natural compounds), interventional therapies (sclerotherapy and various surgical therapies) [168], and lifestyle changes (regular exercise, avoidance of prolonged sitting/standing, weight loss) [178,179].

The pharmacotherapy of CVD includes a couple of established venoactive drugs (suledoxide, calcium dobesilate, pentoxifylline, aspirin), as well as numerous phytochemicals, the most studied being flavonoids (mainly diosmin, quercetin, rutosides), saponosides (mainly escin, horse chestnut seed extract, ruscus extract), saponins from plants (Ginkgo biloba, blueberry and grape seed extracts), catechin (Green tea), and Daflon (an oral micronized venotonic containing 90% diosmin and 10% hesperidin)). Their pleiotropic benefits in CVD, including the mechanisms underlying the antioxidant effect, have been recently summarized by several excellent reviews (see refs. [178,180,181,182,183,184]), which also include comprehensive summative tables. Important, the antioxidant compounds in nutraceuticals also act as antiobesity molecules [185], as well as anti-inflammatory compounds [186].

Sodium–glucose cotransporter 2 inhibitors (SGLT2i) are novel antidiabetics that reshaped the therapeutics of patients with type 2 diabetes are game-changers in an increasing number of non-diabetic pathologies [187,188] via complex, partially elucidated cellular and mitochondrial pathways [189]. Notably, in addition to their glucose-lowering effects, these medications demonstrate multiple beneficial pleiotropic effects in non-diabetic patients with cardiovascular pathologies [190] and chronic kidney disease [191]. In a very recent elegant study, the group of Schini-Kerth demonstrated the presence of SGLT2 expression in both human internal thoracic artery (endothelium and vascular smooth muscle) and coronary microcirculation. The pro-inflammatory cytokines (IL-1ß, IL-6, and TNF-α) enhanced SGLT2 expression in endothelial cells. This promoted oxidative stress, which resulted in endothelial dysfunction and the feedforward of both a pro-inflammatory response (NF-kB activation) and prooxidative response via the AT1 receptor/NADPH oxidase-eNOS signaling pathway [192]. Whether the expression is also present in the venous wall and mediates the crosstalk oxidative stress-inflammation has not been investigated.

Along these lines, we recently demonstrated that acute ex vivo treatment with SGLT2i (empagliflozin or dapagliflozin) of human atrial tissue samples harvested from non-diabetic patients with all types of heart failure undergoing open-heart surgery resulted in a substantial decrease in oxidative stress, along with a reduction in the expression of both isoforms of monoamine oxidase, MAO-A and MAO-B, mitochondrial enzymes that catabolize catecholamines and serotonin [193]. It has also been reported that MAOs are potent regulators of chronic inflammation in various pathologies, including obesity related-cardiovascular diseases (for a recent excellent review see ref. [194]). As such, the contribution of MAO, whose expression has been reported to increase with aging [195,196], to the local oxidative stress in the local varices is an uncharted area that requires further investigation.

In patients with cardiometabolic pathologies, a phenomenon denominated pro-vascular ‘regenerative cell exhaustion’, characterized by low content of pro-vascular progenitor cells, monocyte polarization towards a pro-inflammatory phenotype, and impaired vessel repair capacity, has been reported [197]. The combination of SGLT2 inhibitors and GLP-1 receptor agonists has been suggested to provide additive effects in reducing oxidative stress, which could enhance vascular repair, thus mitigating vascular comorbidities linked to obesity and type 2 diabetes [198]. Whether their combination might reverse abnormalities in the venous bed in the early stages of CVD has not been investigated so far.

## 5. Future Research Directions

Homeostasis is preserved by the enhanced autophagic clearance of damaged proteins and organelles (e.g., mitophagy), while the accumulation of such damaged cellular components results in disease generation/progression. Given that antioxidants suppress both beneficial and detrimental ROS, enhancing autophagy may represent a more effective approach to disease management. Currently there is an increasing interest in elucidating the role of dysregulated autophagy in the endothelial cells [199,200] and the complex pathophysiological role of autophagy in vascular remodeling has been recently reviewed together with the autophagy-targeted pharmacological agents [201]; these molecules might be of interest in counteracting the venous wall remodeling in the setting of CVD.

Novel research techniques may be used to dissect the pathomechanism of CVD at molecular level. Recently, a minimally invasive technique has become available to sample freshly isolated human ECs (FIHECs) by means of an atraumatic J-shaped wire that is passed through an angiocatheter placed in a forearm vein [202]. FIHECs adhere to the wire and are further isolated and studied; this approach might be useful for the phenotypic and functional characterization of the endothelial cells harvested from the VV.

Also, there is an increased interest in the characterization of endothelial cell heterogeneity along the different vascular beds and plasticity, i.e., the adaptive genetic, functional, and structural alterations in diseases [203], and also the sex- and age-dependent heterogeneity, by using high-throughput sequencing technologies (scRNA-seq) [204] and metabolomics [205]; mapping transcriptional changes within the diseased venous wall and identifying circulating biomarkers by targeted metabolomics might provide useful insights into the disease dynamics.

## 6. Conclusions

Oxidative stress is a pathophysiological mechanism that contributes to the progression of CVD, both locally at the level of varicose veins, in its early stages and systemically, in the peripheral venous blood, in the late stages. The studies that demonstrated either the increased oxidants or decreased antioxidants were small-sized and non-randomized, employing different methodologies and biomarkers to assess the oxidative phenotype in patients where the presence/severity of obesity, the most important comorbidity, was not reported.

The persistent gaps in understanding the pathogenesis and progression of chronic venous disease, in particular the stage-dependent contribution of various sources of oxidative stress, its specific targets, and links with the chronic sterile inflamation, require ongoing research. Elucidating the pathogenesis of redox impairment in CVD, despite being hampered by complexity of the in vivo oxidative stress biochemistry and methodology limitations, remains an active field of research in order to identify efficient pharmacological strategies. By employing modern integrative methods, currently used in systems biology and multi-omics research, it may be feasible to illuminate these interactions both at local and systemic levels, pinpointing specific cellular and molecular sites of the damage in relation to the CVD stages.

## Figures and Tables

**Table 1:** Summary of clinical studies assessing ROS generation and their enzymatic sources in CVD.

Study Groups	Samples Assessed	Relevant Findings	Ref.
**ROS Generation**			
14 VV patients (11♀:3♂) *vs.* 15 controls (11♀:4♂)	Proximal and distal GSV samples harvested during VV surgery	VVs generate significantly more superoxide anion than healthy veins in their distal segments (*p* < 0.001) Sources of superoxide anion: NADPH oxidases and uncoupled NOS	[67]
30 patients (21♀:9♂) with CEAP C_2_ *vs.* 30 controls (20♀:10♂)	Peripheral venous blood from a dorsal foot vein of the involved leg after one-hour orthostatism, prior to GSV stripping and in same circumstances, one month after stripping	Local ROMs were significantly higher in pre-surgery patients *vs.* control (*p* < 0.0001) Local ROMs after 30 days post-surgery were significantly decreased *vs.* pre-surgery in patients (*p* < 0.0001), while their level in controls was unmodified	[68]
96 patients with CEAP C_2_ (69♀:27♂): 54 unilateral venous reflux (U-CVD) (39♀:15♂) and 42 with bilateral venous reflux (B-CVD) (30♀:12♂) *vs.* 65 controls (46♀:19♂)	Peripheral venous blood	No statistical difference in the ROM levels among U-CVD, B-CVD and controls	[69]
8 VV patients CEAP C_2_ (2♀:6♂)	Varicose vein blood *vs.* peripheral venous blood	Peroxides concentration in erythrocytes membrane was similar in the blood from VVs and peripheral (antecubital) vein	[70]
28 patients with CVD (12 obese/OB, 16 non-obese/non-OB)	VV samples from both obese and non-obese patients undergoing cryostripping surgery for varicose vein ablation	Increased levels of superoxide and hydrogen peroxide in VVs from OB *vs.* non-OB patients with CVD (*p* < 0.05)	[72]
**Myeloperoxidase (MPO)**			
36 VV patients complicated or not with superficial thrombophlebitis (21♀:15♂) *vs.* 6 normal veins—cadaver organ procurement (2♀:4♂)	GSV samples harvested during surgery: VVs with superficial thrombophlebitis (STP) and unchanged veins (UV) *vs.* normal veins	The highest MPO content was found in the VVs, followed by STP samples	[77]
9 VV patients (5♀:4♂) CEAP C_2_ *vs.* 10 controls (5♀:5♂)	Peripheral venous blood	No statistical difference in plasma activity of MPO in patients *vs.* controls	[78]
**Xanthine oxidase (XO)**			
36 VV patients complicated or not with superficial thrombophlebitis (21♀:15♂) *vs.* 6 normal veins—cadaver organ procurement (2♀:4♂)	GSV samples harvested during VV surgery—VVs with superficial thrombophlebitis and UV *vs.* normal veins	Positive intense staining for XO of the *endothelium* in both VVs and VVs with superficial thrombophlebitis Positive intense staining for XO also of the *vasa vasorum* in VVs with superficial thrombophlebitis	[77]
9 VV patients (5♀:4♂) CEAP C_2_ *vs.* 10 controls (5♀:5♂)	Peripheral venous blood	Plasma XO activity not statistically different in patients *vs.* controls	[78]

Abbreviations: CEAP—Clinical, Etiological, Anatomical and Pathophysiological Classification of chronic venous disease (CVD); GSV—great saphenous vein; NOS—nitric oxide synthases; ROMs, reactive oxygen metabolites; ROS—reactive oxygen species; VVs—varicose veins.

**Table 2:** Summary of clinical studies assessing oxidative stress biomarkers in CVD.

Study Groups	Samples Assessed	Relevant Findings	Ref.
**Malondialdehyde (MDA) / Thiobarbituric Acid Reactive Substances (TBARS)**			
31 VV patients CEAP C_2/3_ (23♀:8♂)	VV blood samples harvested during VV surgery + peripheral venous blood from the same patients	MDA concentration was significantly higher in the VV blood samples *vs.* peripheral blood in females (*p* < 0.007) but not in males (*p* > 0.05)	[83]
31 VV patients CEAP C_2/3_ (23♀:8♂) *vs.* 31 controls (20♀:11♂)	Peripheral venous blood and VVs blood *vs.* peripheral venous blood	Overall MDA concentrations were significantly higher in venous blood plasma of CVD patients than in controls (*p* < 0.0005) MDA levels were significantly higher in obese patients *vs.* patients with normal BMI (*p* < 0.001)	[84]
31 VV patients CEAP C_2_ (23♀:8♂)	GSV samples harvested during VV surgery: insufficient proximal GSVs, sufficient segments of distal GSVs and varicose saphenous tributaries	The highest MDA concentration was found in VVs and insufficient veins	[85]
35 CVI patients (24♀:11♂) subdivided in 4 groups: M (mild symptoms)—12, S (severe symptoms)—23, Group I (< 10 years of evolution)—15, Group II (>10 years of evolution)—20 *vs.* 23 controls (16♀:7♂)	Peripheral venous blood	MDA concentration in patients was significantly higher in all diseased groups *vs.* controls Higher MDA levels were reported in female *vs.* males with CVI	[86]
9 VV patients CEAP C_2_ (5♀:4♂) *vs.* 10 controls (5♀:5♂)	Peripheral venous blood	MDA concentration was significantly higher in VV blood *vs.* peripheral blood in females (but not in males)	[78]
10 ♂ VV patients *vs.* 10 ♂ controls *vs.* 10 ♂ abdominal aortic aneurysm (AAA) group	GSV samples harvested during VV surgery + peripheral venous blood *vs.* Peripheral venous blood *vs.* Aneurysmal sac samples harvested during surgical repair	MDA concentration in tissue homogenates from ***VV*** was high and comparable to the AAA samples MDA concentration in ***plasma*** of VV patients significantly lower than in the AAA group MDA concentration in plasma of VV patients—slight increase as compared to controls	[88]
44 CVI ♀ with 2 subgroups: Moderate CVI (CEAP C_2/3_) = 26 Severe CVI (CEAP C_4/5/6_) = 18 *vs.* 25 ♀ controls	Peripheral venous blood	Elevated plasma levels of MDA in CVI *vs.* healthy women, which tend to increase with the severity of the disease	[87]
52 VV patients CEAP C_2_ (35♀:17♂) *vs.* 52 controls (36♀:16♂)	Great Saphenous Vein (GSV) samples harvested during varicose vein surgery *vs.* Healthy GSV samples harvested for CABG patients	Significantly higher MDA in VV patients *vs.* controls	[89]
110 CVI patients—2 groups: 81 with reflux (R group) with 2 subgroups (<50y—32, ≥50y—49) and 29 with no reflux (NR group) with 2 subgroups (<50y—13, ≥50y—16)	Peripheral venous blood	MDA plasma levels were significantly higher in the R group *vs.* NR group (*p* < 0.05) Patients R < 50 exhibited the highest significant MDA increase among the 4 subgroups (*p* < 0.005)	[73]
37 CVI (C_2_–C_4_) patients with 2 subgroups: 32 CVI and 5 CVI + T2DM (32♀:5♂) *vs.* 25 controls (18♀:7♂)	Peripheral venous blood	Increased MDA levels in CVI (*p* < 0.003) and CVI + T2DM (*p* < 0.004) *vs.* controls MDA levels were higher in the CVI + T2DM than in the CVI group (*p* < 0.003)	[103]
25 VV patients CEAP C_2_ (11♀:14♂) *vs.* 25 controls (10♀:15♂)	Peripheral venous blood	No significant difference in MDA between the groups	[90]
36 VV patients complicated or not with superficial thrombophlebitis (21♀:15♂) *vs.* 6 controls during cadaver organ procurement (2♀:4♂)	GSV samples harvested during VV surgery: 34 VV, 9 VV with superficial thrombophlebitis, 27 unchanged vein (UV) and competent parts of diseased GSV *vs.* healthy vein samples	TBARS levels in VVs complicated with thrombophlebitis were the highest among the groups TBARS levels were higher in the VVs and UVs *vs.* controls	[77]
31 VV patients CEAP C_2/3_ (23♀:8♂)	GSV samples harvested during VV surgery—insufficient proximal GSVs, sufficient segments of distal GSVs and varicose saphenous tributaries	TBARS levels in the insufficient veins were 4x higher than in sufficient veins (*p* < 0.0001) TBARS levels—higher in VVs and insufficient veins *vs.* control veins (*p* < 0.0001)	[104]
8 VV patients CEAP C_2_ (2♀:6♂)	VV samples harvested during VV surgery + peripheral venous blood	Significant higher level of TBARS in VV plasma as compared to peripheral vein plasma (*p* < 0.05)	[70]
23 VV patients (14♀:9♂)	GSV samples harvested during VV surgery: VV samples, normal GSV samples and thrombosed samples	TBARS levels in VVs—significantly decreased *vs.* normal GSV (*p* < 0.05) TBARS in VV with thrombophlebitis—increased compared to normal veins (*p* < 0.05)	[91]
**Protein carbonyls**			
9 VV patients CEAP C_2_ (5♀:4♂) *vs.* 10 controls (5♀:5♂)	Peripheral venous blood	Plasma carbonyls—significantly higher (almost double) in VV patients *vs.* controls (*p* < 0.001)	[78]
8 VV patients CEAP C_2_ (2♀:6♂)	VV samples harvested during VV surgery + peripheral venous blood	Higher levels of protein carbonyls in VV plasma as compared to peripheral vein plasma (*p* < 0.05) Higher levels of protein carbonyls in erythrocyte membrane isolated from the VV blood *vs.* peripheral vein blood (*p* < 0.05)	[70]
**Advanced oxidation protein products (AOPP), Total 3-nitrotyrosine (3-NT)**			
9 VV patients CEAP C_2_ (5♀:4♂) *vs.* 10 controls (5♀:5♂)	Peripheral venous blood	AOPP and 3-NT—no statistical difference in VV patients *vs.* controls	[78]
**Prolidase enzyme**			
30 VV patients CEAP C_2_ (13♀:17♂) *vs.* Control 1 group (30—12♀:18♂) *vs.* Control 2 group (20—16♀:14♂)	VVs harvested during VV surgery + peripheral venous blood *vs.* Peripheral venous blood *vs.* Healthy GSV samples harvested for CABG	Prolidase enzyme activity in tissue was higher in VVs *vs.* control 2 group (*p* < 0.008) No significant difference in serum prolidase enzyme activity *vs.* control 1 group	[102]

Abbreviations: BMI—body mass index; CABG—coronary artery bypass graft; CEAP classification—Clinical, Etiologic, Anatomic and Pathophysiologic Classification; CVD—chronic venous disease; CVI—chronic venous insufficiency; GSV—great saphenous vein; T2DM—type 2 diabetes mellitus; VVs—varicose veins.

**Table 3:** Summary of clinical studies that assessed antioxidant capacity/power in CVD.

Study Groups	Samples Assessed	Relevant Findings	Ref.
**Total Antioxidant Capacity (TAC)**			
35 CVI patients (24♀:11♂) subdivided in 4 groups: M (mild symptoms)—12 S (severe symptoms)—23 Group I (<10 years of evolution)—15, Group II (>10 years of evolution)—20 *vs.* 23 controls (16♀:7♂)	Peripheral venous blood	TAC was significantly higher in patients *vs.* controls (*p* < 0.05) TAC highest differences were in S group (*p* < 0.016) and group II (*p* < 0.013) *vs.* controls TAC was significantly lower in CVI women *vs.* CVI men (*p* < 0.05) TAC was significantly higher in overweight patients (BMI ≥ 25) *vs.* patients with BMI < 25 (*p* < 0.05) TAC was not influenced by age	[86]
30 VV patients CEAP C_2_ (13♀:17♂) *vs.* Control group 1 (30—12♀:18♂) *vs.* Control group 2 (20—16♀:14♂)	Varicose vein samples harvested during VV surgery + peripheral venous blood Peripheral venous blood Healthy Great Saphenous Vein (GSV) samples harvested for CABG	Tissue TAC level was significantly decrease in VV patients *vs.* control group 2 (*p* = 0.003) No significant difference in plasma TAC level in patients *vs.* control group 1	[102]
65 VV patients CEAP C_2_ (49♀:16♂) *vs.* Control group 1—10 (8♀:2♂) and Control group 2—20 (11♀:9♂)	GSV samples + peripheral venous blood *vs.* Healthy GSVs harvested for CABG and Peripheral venous blood	Tissue TAC level was significantly decreased in patients *vs.* control group 1 (*p* < 0.05) Plasma TAC level was significantly decreased in patients *vs.* control group 2 (*p* < 0.001)	[109]
9 VV patients CEAP C_2_ (5♀:4♂) *vs.* 10 controls (5♀:5♂)	Peripheral venous blood	No significant differences in TAC and ORAC values in patients *vs.* controls	[78]
**Non-Enzymatic Antioxidant Capacity (NEAC)**			
8 VV patients CEAP C_2_ (2♀:6♂)	Peripheral venous blood from antecubital vein and from VVs of the same patient	NEAC was significantly decreased in VV plasma *vs.* peripheral vein plasma (*p* < 0.05)	[70]
**Total antioxidant power (TAP) / Ferric**-**Reducing Ability of Plasma (FRAP)**			
31VV patients CEAP C_2/3_ (23♀:8♂)	Peripheral venous blood from the antecubital vein and from VVs of the same patient	FRAP level was significantly decreased in VV blood *vs.* peripheral blood (*p* < 0.002) FRAP level in women was significantly lower in VV blood *vs.* peripheral blood (*p* < 0.003) No significant differences in FRAP level in male patients in both blood samples	[83]
31 VV patients CEAP C_2_ (23♀:8♂)	GSV samples harvested during VV surgery from: insufficient proximal GSVs, sufficient distal GSVs and varicose veins tributaries to GSVs	FRAP level was statistically significant lower in VVs and insufficient GSV samples *vs.* sufficient GSV samples (*p* < 0.001)	[85]

Abbreviations: CABG—coronary artery bypass graft, CEAP—Clinical, Etiologic, Anatomic and Pathophysiologic Classification; CVI—chronic venous insufficiency; FRAP—ferric-reducing ability of plasma; GSV—great saphenous vein; VVs—varicose veins; ORAC—oxygen radical absorbance capacity; TAP—total antioxidant power.

**Table 4:** Summary of changes in enzymatic antioxidants in CVD.

Study Groups	Samples Assessed	Relevant Findings	Ref.
**Superoxide dismutase (SOD)**			
24 VV patients (20♀:4♂)	VV samples harvested during VV surgery: 21 mid-thigh GSVs, 23 distal calf varicosities	SOD activity—significantly higher in the distal calf varicosities *vs.* mid-thigh GSV walls (*p* < 0.05)	[113]
31 VV patients CEAP C_2/3_ (23♀:8♂)	GSV segments harvested during VV surgery from the same patient: insufficient proximal GSVs, sufficient distal GSVs and varicose saphenous tributaries	SOD activity—significantly higher in insufficient GSVs and varices *vs.* sufficient GSV segments (*p* < 0.0001) Increased iron content in the same samples responsible for DNA oxidative damage	[104]
65 VV patients CEAP C_2_ (49F:16M)	GSV samples harvested during VV surgery and peripheral venous blood (plasma and erythrocytes)	SOD activity in ***tissue***—significantly *increased* in patients *vs.* control (*p* < 0.05) SOD activity *in **erythrocytes***—significantly *decreased* in patients *vs.* control (*p* < 0.001)	[109]
23 VV patients (14F:9M)	GSV samples harvested during VV surgery: segments of VV, of veins with thrombophlebitis, and of normal veins	SOD activity—significantly decreased in VV *vs.* normal veins (*p* < 0.05) and unchanged in VV with thrombophlebitis	[91]
37 CVI patients—two subgroups: 32 CEAP C_2_-C_4_; 5 CEAP C_2_ + T2DM (32♀:5♂) *vs. * 25 controls (18♀:7♂)	Peripheral venous blood	SOD activity—significantly decreased *vs.* control in both CEAP C_2_-C_4_ patients and CEAP C_2_ diabetic patients (*p* < 0.05)	[103]
10 VV patients (9♀:1♂) *vs.* 4 controls (1♀:3♂) *vs.* 6 trauma patients (6♂)	GSV samples harvested during VV surgery *vs.* Healthy GSV samples harvested for CABG *vs. * Healthy veins	SOD level—significantly decreased in VVs *vs.* controls (*p* < 0.0001)	[117]
52 VV patients CEAP C_2_ (35♀:17♂) *vs.* 52 controls (36♀:16♂)	GSV samples Healthy GSV samples harvested for CABG	SOD activity—no statistically significant difference in VV patients *vs.* controls	[89]
**Glutathione peroxidase (GPx)**			
31 VV patients CEAP C_2_ (23♀:8♂)	GSV samples harvested during VV surgery: insufficient proximal GSV, sufficient segments of distal GSV and varicose saphenous tributaries	GPx tissue activity—significantly higher in varices and insufficient veins *vs.* sufficient veins (*p* < 0.001)	[104]
37 CVI patients—two subgroups: 32 CEAP C_2_-C_4_; 5 CVI + T2DM (32♀:5♂) *vs. * 25 controls (18♀:7♂)	Peripheral venous blood	GPx plasma activity—significantly lower in CVI and CVI + T2DM *vs.* controls (*p* < 0.05)	[103]
52 VV patients CEAP C_2_ (35♀:17♂) *vs.* 52 controls (36♀:16♂)	GSV samples harvested during VV surgery *vs.* Healthy GSV samples harvested for CABG	No statistically significant difference in GPx in patients *vs.* controls	[89]
**Glutathione S-transferase (GST)**			
52 VV patients CEAP C_2_ (35♀:17♂) *vs.* 52 controls (36♀:16♂)	GSV samples harvested during VV surgery *vs.* Healthy GSV samples harvested for CABG	No statistically significant difference in GST in patient *vs.* controls	[89]
**Glutathione-reductase (GR)**			
23 VV patients (14♀:9♂)	GSV samples harvested during VV surgery: segments of VV, of veins with thrombophlebitis, and of normal veins	No statistically significant difference in GR in diseased GSV samples *vs.* normal GSV	[91]
**Catalase (CAT)**			
52 VV patients CEAP C_2_ (35♀:17♂) *vs.* 52 controls (36♀:16♂)	GSV samples harvested during VV surgery Healthy GSV samples harvested for CABG	CAT activity—significantly increased in patients *vs.* control (*p* < 0.001)	[89]
9 VV patients CEAP C_2_ (5♀:4♂) *vs.* 10 controls (5♀:5♂)	Peripheral venous blood	CAT activity—significantly lower in patients *vs.* control (*p* < 0.001)	[78]
37 CVI patients—two subgroups: 32 CEAP C_2_-C_4_; 5 CVI + T2DM (32♀:5♂) *vs. * 25 controls (18♀:7♂)	Peripheral venous blood	CAT activity—significantly decreased in CVI and CVI + T2DM *vs.* control (*p* < 0.05)	[103]
8 VV patients CEAP C_2_ (2♀:6♂)	Peripheral venous blood from the antecubital vein and from VVs of same patient	CAT activity—significantly lower in VV hemolysate *vs.* peripheral vein hemolysate (*p* < 0.05)	[70]

Abbreviations: CABG—coronary artery bypass graft; CEAP—Clinical, Etiologic, Anatomic and Pathophysiologic Classification; CVD—chronic venous disease; CVI—chronic venous insufficiency; GSV—great saphenous vein; VVs—varicose veins.

**Table 5:** Summary of changes in non-enzymatic antioxidants in CVD.

Study Groups	Samples Assessed	Relevant Findings	Ref.
**Glutathione (GSH)**			
9 VV patients CEAP C_2_ (5♀:4♂) *vs.* 10 controls (5♀:5♂)	Peripheral venous blood	Trend of decreased GSH in VV patients but no statistical difference *vs.* controls	[78]
65 VV group CEAP C_2_ (49♀:16♂) *vs.* Control 1 group (10—8♀:2♂) *vs.* Control 2 group (20—11♀:9♂)	Great Saphenous Vein (GSV) samples harvested during VV surgery + peripheral venous blood *vs.* Healthy GSV samples harvested for CABG *vs.* Peripheral venous blood from healthy individuals	No significant changes in GSH concentration in tissue (venous wall) and plasma from VV patients *vs.* controls	[109]
37 CVI patients—two subgroups: 32 CEAP C_2_-C_4_; 5 CVI + T2DM (32♀:5♂) *vs.* 25 controls (18♀:7♂)	Peripheral venous blood	GSH level—significantly lower in CVI diabetics *vs.* controls (*p* < 0.004) GSH level—significantly lower in CVI diabetics *vs.* CVI patients (*p* < 0.05)	[103]
8 VV patients CEAP C_2_ (2♀:6♂)	Peripheral venous blood from antecubital vein and VV blood from the same patient	No statistical difference in GSH from erythrocytes, plasma in VV blood *vs.* peripheral vein blood	[70]
**Thiols**			
9 VV patients CEAP C_2_ (5♀:4♂) *vs.* 10 controls (5♀:5♂)	Peripheral venous blood	Total thiols—significantly *lower* in patient *vs.* control group	[78]
8 VV patients CEAP C_2_ (2♀:6♂)	Peripheral venous blood from the antecubital vein and from VVs of the same patient used to prepare: plasma, hemolysate and erythrocyte membranes	Thiols—significantly lower in products from the VVs blood *vs.* those from peripheral vein blood (*p* < 0.05)	[70]
10 VV patients (9♂:1♀) *vs.* 4 controls from vascular bypass *vs.* 6 controls from trauma patients	VV samples harvested during surgery *vs.* Healthy GSV samples harvested for CABG *vs.* Healthy vein samples	Total thiols—significantly higher in VV patients *vs.* both controls (*p* < 0.0001)	[117]
**Uric Acid (UA)**			
35 CVI patients (24♀:11♂) in 2 subgroups: - with mild symptoms (M group)—12 and severe symptoms (S group)—23 - with <10 years of evolution (group I)—15, >10 years of evolution (group II)—20 *vs.* 23 controls (16♀:7♂)	Peripheral venous blood	UA—significantly lower in M group (*p* < 0.047) and group I (*p* < 0.034) *vs.* controls UA—significantly lower in CVI women *vs.* female control group (*p* < 0.014)	[86]
9 VV patients CEAP C_2_ (5♀:4♂) *vs.* 10 controls (5♀:5♂)	Peripheral venous blood	No significant differences in UA levels in VV patients *vs.* controls	[78]
***Ascorbic acid***			
23 VV patients (14♀:9♂)	GSV samples harvested during VV surgery: VVs, thrombosed segments or normal GSV segments	Ascorbic acid—significantly decreased in VVs *vs.* normal GSV samples (*p* < 0.05) Ascorbic acid—significantly increased in VV with thrombophlebitis *vs.* normal GSV samples (*p* < 0.05)	[91]

Abbreviations: CABG—coronary artery bypass graft; CEAP—Classification of Chronic Venous Disease/CVD—Clinical, Etiologic, Anatomic and Pathophysiologic Classification; CVI—chronic venous insufficiency; T2DM—Type 2 diabetes mellitus; GSV—great saphenous vein; VVs—varicose veins.

## Data Availability

The article is a narrative review. No new data were generated or analyzed.

## References

[1] Bergan J.J., Schmid-Schönbein G.W., Smith P.D., Nicolaides A.N., Boisseau M.R., Eklof B. (2006). Chronic venous disease. N. Engl. J. Med..

[2] Davies A.H. (2019). The Seriousness of Chronic Venous Disease: A Review of Real-World Evidence. Adv. Ther..

[3] Salim S., Machin M., Patterson B.O., Onida S., Davies A.H. (2021). Global Epidemiology of Chronic Venous Disease: A Systematic Review with Pooled Prevalence Analysis. Ann. Surg..

[4] Ortega M.A., Fraile-Martínez O., García-Montero C., Álvarez-Mon M.A., Chaowen C., Ruiz-Grande F., Pekarek L., Monserrat J., Asúnsolo A., García-Honduvilla N. (2021). Understanding Chronic Venous Disease: A Critical Overview of Its Pathophysiology and Medical Management. J. Clin. Med..

[5] Nicolaides A.N., Labropoulos N. (2019). Burden and Suffering in Chronic Venous Disease. Adv. Ther..

[6] Finkelstein E.A., Khavjou O.A., Thompson H., Trogdon J.G., Pan L., Sherry B., Dietz W. (2012). Obesity and severe obesity forecasts through 2030. Am. J. Prev. Med..

[7] Davies H.O., Popplewell M., Singhal R., Smith N., Bradbury A.W. (2017). Obesity and lower limb venous disease—The epidemic of phlebesity. Phlebology.

[8] Deol Z.K., Lakhanpal S., Franzon G., Pappas P.J. (2020). Effect of obesity on chronic venous insufficiency treatment outcomes. J. Vasc. Surg. Venous Lymphat. Disord..

[9] Michaelidou M., Pappachan J.M., Jeeyavudeen M.S. (2023). Management of diabesity: Current concepts. World J. Diabetes.

[10] Jarošíková R., Roztočil K., Husáková J., Dubský M., Bém R., Wosková V., Fejfarová V. (2023). Chronic Venous Disease and Its Intersections with Diabetes Mellitus. Physiol. Res..

[11] De Maeseneer M.G., Kakkos S.K., Aherne T., Baekgaard N., Black S., Blomgren L., Giannoukas A., Gohel M., de Graaf R., Hamel-Desnos C. (2022). Editor’s Choice—European Society for Vascular Surgery (ESVS) 2022 Clinical Practice Guidelines on the Management of Chronic Venous Disease of the Lower Limbs. Eur. J. Vasc. Endovasc. Surg..

[12] Gloviczki P., Lawrence P.F., Wasan S.M., Meissner M.H., Almeida J., Brown K.R., Bush R.L., Di Iorio M., Fish J., Fukaya E. (2024). The 2023 Society for Vascular Surgery, American Venous Forum, and American Vein and Lymphatic Society clinical practice guidelines for the management of varicose veins of the lower extremities. J. Vasc. Surg. Venous Lymphat. Disord..

[13] Bernatchez S.F., Eysaman-Walker J., Weir D. (2022). Venous Leg Ulcers: A Review of Published Assessment and Treatment Algorithms. Adv. Wound Care.

[14] Labropoulos N. (2019). How Does Chronic Venous Disease Progress from the First Symptoms to the Advanced Stages? A Review. Adv. Ther..

[15] Chung J.H., Heo S. (2024). Varicose Veins and the Diagnosis of Chronic Venous Disease in the Lower Extremities. J. Chest Surg..

[16] Pfisterer L., König G., Hecker M., Korff T. (2014). Pathogenesis of varicose veins—Lessons from biomechanics. Vasa.

[17] Attaran R.R., Carr J.G. (2023). Chronic Venous Disease of the Lower Extremities: A State-of-the Art Review. J. Soc. Cardiovasc. Angiogr. Interv..

[18] Raffetto J.D. (2018). Pathophysiology of Chronic Venous Disease and Venous Ulcers. Surg. Clin. N. Am..

[19] Lim C.S., Gohel M.S., Shepherd A.C., Paleolog E., Davies A.H. (2011). Venous hypoxia: A poorly studied etiological factor of varicose veins. J. Vasc. Res..

[20] Costa D., Andreucci M., Ielapi N., Serraino G.F., Mastroroberto P., Bracale U.M., Serra R. (2023). Molecular Determinants of Chronic Venous Disease: A Comprehensive Review. Int. J. Mol. Sci..

[21] Ojdana D., Safiejko K., Lipska A., Sacha P., Wieczorek P., Radziwon P., Dadan J., Tryniszewska E. (2009). The inflammatory reaction during chronic venous disease of lower limbs. Folia Histochem. Cytobiol..

[22] Sies H., Berndt C., Jones D.P. (2017). Oxidative Stress. Annu. Rev. Biochem..

[23] Singh N., Dhalla A.K., Seneviratne C., Singal P.K. (1995). Oxidative stress and heart failure. Mol. Cell Biochem..

[24] Guzik T.J., Touyz R.M. (2017). Oxidative Stress, Inflammation, and Vascular Aging in Hypertension. Hypertension.

[25] Simantiris S., Papastamos C., Antonopoulos A.S., Theofilis P., Sagris M., Bounta M., Konisti G., Galiatsatos N., Xanthaki A., Tsioufis K. (2023). Oxidative Stress Biomarkers in Coronary Artery Disease. Curr. Top. Med. Chem..

[26] Pfenniger A., Yoo S., Arora R. (2024). Oxidative stress and atrial fibrillation. J. Mol. Cell Cardiol..

[27] Muntean D.M., Sturza A., Dănilă M.D., Borza C., Duicu O.M., Mornoș C. (2016). The Role of Mitochondrial Reactive Oxygen Species in Cardiovascular Injury and Protective Strategies. Oxid. Med. Cell Longev..

[28] Chen Q., Wang Q., Zhu J., Xiao Q., Zhang L. (2018). Reactive oxygen species: Key regulators in vascular health and diseases. Br. J. Pharmacol..

[29] Abrashev H., Abrasheva D., Nikolov N., Ananiev J., Georgieva E. (2025). A Systematic Review of Endothelial Dysfunction in Chronic Venous Disease—Inflammation, Oxidative Stress, and Shear Stress. Int. J. Mol. Sci..

[30] Pocock E.S., Alsaigh T., Mazor R., Schmid-Schönbein G.W. (2014). Cellular and molecular basis of Venous insufficiency. Vasc. Cell.

[31] Ortega M.A., Fraile-Martínez O., García-Montero C., Pekarek L., Alvarez-Mon M.A., Guijarro L.G., Del Carmen Boyano M., Sainz F., Álvarez-Mon M., Buján J. (2021). Tissue remodelling and increased DNA damage in patients with incompetent valves in chronic venous insufficiency. J. Cell Mol. Med..

[32] Gwozdzinski L., Pieniazek A., Gwozdzinski K. (2024). Factors Influencing Venous Remodeling in the Development of Varicose Veins of the Lower Limbs. Int. J. Mol. Sci..

[33] Gwozdzinski L., Pieniazek A., Gwozdzinski K. (2024). The Roles of Oxidative Stress and Red Blood Cells in the Pathology of the Varicose Vein. Int. J. Mol. Sci..

[34] Saberianpour S., Modaghegh M.H.S., Rahimi H., Kamyar M.M. (2021). Role of mechanosignaling on pathology of varicose vein. Biophys. Rev..

[35] Raffetto J.D., Ligi D., Maniscalco R., Khalil R.A., Mannello F. (2020). Why Venous Leg Ulcers Have Difficulty Healing: Overview on Pathophysiology, Clinical Consequences, and Treatment. J. Clin. Med..

[36] Lyons O.T.A., Saha P., Smith A. (2020). Redox dysregulation in the pathogenesis of chronic venous ulceration. Free Radic. Biol. Med..

[37] Sies H., Jones D.P. (2020). Reactive oxygen species (ROS) as pleiotropic physiological signalling agents. Nat. Rev. Mol. Cell Biol..

[38] Brandes R.P., Weissmann N., Schröder K. (2010). NADPH oxidases in cardiovascular disease. Free Radic. Biol. Med..

[39] Drummond G.R., Sobey C.G. (2014). Endothelial NADPH oxidases: Which NOX to target in vascular disease?. Trends Endocrinol. Metab..

[40] Lassègue B., Griendling K.K. (2010). NADPH Oxidases: Functions and Pathologies in the Vasculature. Arter. Thromb. Vasc. Biol..

[41] Vermot A., Petit-Härtlein I., Smith S.M.E., Fieschi F. (2021). NADPH Oxidases (NOX): An Overview from Discovery, Molecular Mechanisms to Physiology and Pathology. Antioxidants.

[42] Burtenshaw D., Hakimjavadi R., Redmond E.M., Cahill P.A. (2017). Nox, Reactive Oxygen Species and Regulation of Vascular Cell Fate. Antioxidants.

[43] Konior A., Schramm A., Czesnikiewicz-Guzik M., Guzik T.J. (2014). NADPH oxidases in vascular pathology. Antioxid. Redox Signal.

[44] Tejero J., Shiva S., Gladwin M.T. (2019). Sources of Vascular Nitric Oxide and Reactive Oxygen Species and Their Regulation. Physiol. Rev..

[45] Battelli M.G., Polito L., Bortolotti M., Bolognesi A. (2016). Xanthine Oxidoreductase-Derived Reactive Species: Physiological and Pathological Effects. Oxid. Med. Cell Longev..

[46] Miller A.F. (2012). Superoxide dismutases: Ancient enzymes and new insights. FEBS Lett..

[47] Davidson S.M., Duchen M.R. (2007). Endothelial Mitochondria. Circ. Res..

[48] Di Lisa F., Kaludercic N., Carpi A., Menabò R., Giorgio M. (2009). Mitochondria and vascular pathology. Pharmacol. Rep..

[49] Grossini E., Venkatesan S., Ola Pour M.M. (2025). Mitochondrial Dysfunction in Endothelial Cells: A Key Driver of Organ Disorders and Aging. Antioxidants.

[50] Therade-Matharan S., Laemmel E., Duranteau J., Vicaut E. (2004). Reoxygenation after hypoxia and glucose depletion causes reactive oxygen species production by mitochondria in HUVEC. Am. J. Physiol. Regul. Integr. Comp. Physiol..

[51] Onukwufor J.O., Berry B.J., Wojtovich A.P. (2019). Physiologic Implications of Reactive Oxygen Species Production by Mitochondrial Complex I Reverse Electron Transport. Antioxidants.

[52] Hernansanz-Agustín P., Enríquez J.A. (2021). Generation of Reactive Oxygen Species by Mitochondria. Antioxidants.

[53] Pearlstein D.P., Ali M.H., Mungai P.T., Hynes K.L., Gewertz B.L., Schumacker P.T. (2002). Role of mitochondrial oxidant generation in endothelial cell responses to hypoxia. Arter. Thromb. Vasc. Biol..

[54] Ali M.H., Pearlstein D.P., Mathieu C.E., Schumacker P.T. (2004). Mitochondrial requirement for endothelial responses to cyclic strain: Implications for mechanotransduction. Am. J. Physiol. Lung Cell Mol. Physiol..

[55] Sies H. (2017). Hydrogen peroxide as a central redox signaling molecule in physiological oxidative stress: Oxidative eustress. Redox Biol..

[56] Ostadkarampour M., Putnins E.E. (2021). Monoamine Oxidase Inhibitors: A Review of Their Anti-Inflammatory Therapeutic Potential and Mechanisms of Action. Front. Pharmacol..

[57] Sturza A., Popoiu C.M., Ionică M., Duicu O.M., Olariu S., Muntean D.M., Boia E.S. (2019). Monoamine Oxidase-Related Vascular Oxidative Stress in Diseases Associated with Inflammatory Burden. Oxid. Med. Cell Longev..

[58] Sturza A., Muntean D., Crețu O. (2021). Monoamine Oxidase, Obesity and Related Comorbidities: Discovering Bonds. Cellular and Biochemical Mechanisms of Obesity.

[59] Camici G.G., Cosentino F., Tanner F.C., Lüscher T.F. (2008). The role of p66Shc deletion in age-associated arterial dysfunction and disease states. J. Appl. Physiol..

[60] Di Lisa F., Giorgio M., Ferdinandy P., Schulz R. (2017). New aspects of p66Shc in ischaemia reperfusion injury and other cardiovascular diseases. Br. J. Pharmacol..

[61] Sun Y., Lu Y., Saredy J., Wang X., Drummer Iv C., Shao Y., Saaoud F., Xu K., Liu M., Yang W.Y. (2020). ROS systems are a new integrated network for sensing homeostasis and alarming stresses in organelle metabolic processes. Redox Biol..

[62] Daiber A., Di Lisa F., Oelze M., Kröller-Schön S., Steven S., Schulz E., Münzel T. (2017). Crosstalk of mitochondria with NADPH oxidase via reactive oxygen and nitrogen species signalling and its role for vascular function. Br. J. Pharmacol..

[63] Janaszak-Jasiecka A., Płoska A., Wierońska J.M., Dobrucki L.W., Kalinowski L. (2023). Endothelial dysfunction due to eNOS uncoupling: Molecular mechanisms as potential therapeutic targets. Cell Mol. Biol. Lett..

[64] Burtenshaw D., Kitching M., Redmond E.M., Megson I.L., Cahill P.A. (2019). Reactive Oxygen Species (ROS), Intimal Thickening, and Subclinical Atherosclerotic Disease. Front. Cardiovasc. Med..

[65] Guzik T.J., Schramm A., Czesnikiewicz-Guzik M., Laher I. (2014). Functional Implications of Reactive Oxygen Species (ROS) in Human Blood Vessels. Systems Biology of Free Radicals and Antioxidants.

[66] Higashi Y. (2022). Roles of Oxidative Stress and Inflammation in Vascular Endothelial Dysfunction-Related Disease. Antioxidants.

[67] Guzik B., Chwała M., Matusik P., Ludew D., Skiba D., Wilk G., Mrowiecki W., Batko B., Cencora A., Kapelak B. (2011). Mechanisms of increased vascular superoxide production in human varicose veins. Pol. Arch. Med. Wewn..

[68] Flore R., Santoliquido A., Antonio D.L., Pola E., Flex A., Pola R., Muzi M.G., Farinon A., Rulli F., Gaetani E. (2003). Long saphenous vein stripping reduces local level of reactive oxygen metabolites in patients with varicose disease of the lower limbs. World J. Surg..

[69] Flore R., Ponziani F.R., Gerardino L., Santoliquido A., Di Giorgio A., Lupascu A., Nesci A., Tondi P. (2015). Biomarkers of low-grade inflammation in primary varicose veins of the lower limbs. Eur. Rev. Med. Pharmacol. Sci..

[70] Gwozdzinski L., Pieniazek A., Bernasinska-Slomczewska J., Hikisz P., Gwozdzinski K. (2021). Alterations in the Plasma and Red Blood Cell Properties in Patients with Varicose Vein: A Pilot Study. Cardiol. Res. Pract..

[71] Matei S.C., Matei M., Anghel F.M., Murariu M.S., Olariu S. (2022). Cryostripping-A Safe and Efficient Alternative Procedure in Chronic Venous Disease Treatment. J. Clin. Med..

[72] Rațiu S., Mariș M.I., Furdui-Lința A.V., Stanciu-Lelcu T., Borza C., Olariu S., Bratu T., Sturza A., Muntean D.M. (2025). Vitamin D alleviates oxidative stress in varicose veins: A pilot study in obese and non-obese patients. Mol. Cell. Biochem..

[73] Ortega M.A., Romero B., Asúnsolo Á., Sola M., Álavrez-Rocha M.J., Sainz F., Álavrez-Mon M., Buján J., García-Honduvilla N. (2019). Patients with Incompetent Valves in Chronic Venous Insufficiency Show Increased Systematic Lipid Peroxidation and Cellular Oxidative Stress Markers. Oxid. Med. Cell Longev..

[74] Frangie C., Daher J. (2022). Role of myeloperoxidase in inflammation and atherosclerosis (Review). Biomed. Rep..

[75] Sena C.M., Leandro A., Azul L., Seiça R., Perry G. (2018). Vascular Oxidative Stress: Impact and Therapeutic Approaches. Front. Physiol..

[76] Fernandez M.L., Upton Z., Edwards H., Finlayson K., Shooter G.K. (2012). Elevated uric acid correlates with wound severity. Int. Wound J..

[77] Glowinski J., Glowinski S. (2002). Generation of reactive oxygen metabolites by the varicose vein wall. Eur. J. Vasc. Endovasc. Surg..

[78] Condezo-Hoyos L., Rubio M., Arribas S.M., España-Caparrós G., Rodríguez-Rodríguez P., Mujica-Pacheco E., González M.C. (2013). A plasma oxidative stress global index in early stages of chronic venous insufficiency. J. Vasc. Surg..

[79] Lee R., Margaritis M., Channon K.M., Antoniades C. (2012). Evaluating oxidative stress in human cardiovascular disease: Methodological aspects and considerations. Curr. Med. Chem..

[80] Ayala A., Muñoz M.F., Argüelles S. (2014). Lipid peroxidation: Production, metabolism, and signaling mechanisms of malondialdehyde and 4-hydroxy-2-nonenal. Oxid. Med. Cell Longev..

[81] Tsikas D. (2017). Assessment of lipid peroxidation by measuring malondialdehyde (MDA) and relatives in biological samples: Analytical and biological challenges. Anal. Biochem..

[82] De Leon J.A.D., Borges C.R. (2020). Evaluation of Oxidative Stress in Biological Samples Using the Thiobarbituric Acid Reactive Substances Assay. J. Vis. Exp..

[83] Krzyściak W., Kózka M., Kazek G., Stępniewski M. (2009). Selected indicators of the antioxidant systemin the blood of patients with lower limb varicose veins. Acta Angiol..

[84] Kózka M., Krzyściak W., Pietrzycka A., Stepniewski M. (2009). Obesity and its influence on reactive oxygen species (ROS) in the blood of patients with varicose veins of the lower limbs. Przegląd Lek..

[85] Krzyściak W., Kózka M. (2011). Generation of reactive oxygen species by a sufficient, insufficient and varicose vein wall. Acta Biochim. Pol..

[86] Budzyń M., Iskra M., Krasiński Z., Dzieciuchowicz Ł., Kasprzak M., Gryszczyńska B. (2011). Serum iron concentration and plasma oxidant-antioxidant balance in patients with chronic venous insufficency. Med. Sci. Monit..

[87] Budzyń M., Iskra M., Turkiewicz W., Krasiński Z., Gryszczyńska B., Kasprzak M.P. (2018). Plasma concentration of selected biochemical markers of endothelial dysfunction in women with various severity of chronic venous insufficiency (CVI)-A pilot study. PLoS ONE.

[88] Palmieri D., Cafueri G., Mongelli F., Pezzolo A., Pistoia V., Palombo D. (2014). Telomere shortening and increased oxidative stress are restricted to venous tissue in patients with varicose veins: A merely local disease?. Vasc. Med..

[89] Saribal D., Kanber E.M., Hocaoglu-Emre F.S., Akyolcu M.C. (2019). Effects of the oxidative stress and genetic changes in varicose vein patients. Phlebology.

[90] Yasim A., Kilinc M., Aral M., Oksuz H., Kabalci M., Eroglu E., Imrek S. (2008). Serum concentration of procoagulant, endothelial and oxidative stress markers in early primary varicose veins. Phlebology.

[91] Farbiszewski R., Glowinski J., Makarewicz-Plonska M., Chwiecko M., Ostapowicz R., Glowinski S. (1996). Oxygen-Derived Free Radicals as Mediators of Varicose Vein Wall Damage. Vasc. Surg..

[92] Colombo G., Clerici M., Garavaglia M.E., Giustarini D., Rossi R., Milzani A., Dalle-Donne I. (2016). A step-by-step protocol for assaying protein carbonylation in biological samples. J. Chromatogr. B Anal. Technol. Biomed. Life Sci..

[93] Augustyniak E., Adam A., Wojdyla K., Rogowska-Wrzesinska A., Willetts R., Korkmaz A., Atalay M., Weber D., Grune T., Borsa C. (2015). Validation of protein carbonyl measurement: A multi-centre study. Redox Biol..

[94] Selmeci L., Seres L., Antal M., Lukács J., Regöly-Mérei A., Acsády G. (2005). Advanced oxidation protein products (AOPP) for monitoring oxidative stress in critically ill patients: A simple, fast and inexpensive automated technique. Clin. Chem. Lab. Med..

[95] Bagyura Z., Takács A., Kiss L., Dósa E., Vadas R., Nguyen T.D., Dinya E., Soós P., Szelid Z., Láng O. (2022). Level of advanced oxidation protein products is associated with subclinical atherosclerosis. BMC Cardiovasc. Disord..

[96] Wattanapitayakul S.K., Bauer J.A. (2001). Oxidative pathways in cardiovascular disease: Roles, mechanisms, and therapeutic implications. Pharmacol. Ther..

[97] Bodnár E., Bakondi E., Kovács K., Hegedűs C., Lakatos P., Robaszkiewicz A., Regdon Z., Virág L., Szabó É. (2018). Redox Profiling Reveals Clear Differences between Molecular Patterns of Wound Fluids from Acute and Chronic Wounds. Oxid. Med. Cell Longev..

[98] Eni-Aganga I., Lanaghan Z.M., Balasubramaniam M., Dash C., Pandhare J. (2021). PROLIDASE: A Review from Discovery to its Role in Health and Disease. Front. Mol. Biosci..

[99] Isbilen E., Kulaksizoglu S., Kirmizioglu M., Karuserci Komurcu O., Tabur S. (2022). Role of prolidase activity and oxidative stress biomarkers in unexplained infertility. Int. J. Gynaecol. Obs..

[100] Gonullu H., Aslan M., Karadas S., Kati C., Duran L., Milanlioglu A., Aydin M.N., Demir H. (2014). Serum prolidase enzyme activity and oxidative stress levels in patients with acute hemorrhagic stroke. Scand. J. Clin. Lab. Investig..

[101] Aslan M., Duzenli U., Esen R., Soyoral Y.U. (2017). Serum prolidase enzyme activity in obese subjects and its relationship with oxidative stress markers. Clin. Chim. Acta.

[102] Akar İ., İnce İ., Aslan C., Benli İ., Demir O., Altındeger N., Dogan A., Ceber M. (2018). Oxidative Stress and Prolidase Enzyme Activity in the Pathogenesis of Primary Varicose Veins. Vascular.

[103] Karamalakova Y.D., Abrashev H.M., Nikolova G.D., Kavrakov T.T., Gadjeva V.G. (2019). Generation of plasmatic oxidative damages in patients with chronic venous insufficiency. Bulg. Chem. Commun..

[104] Krzyściak W., Kowalska J., Kózka M., Papież M.A., Kwiatek W.M. (2012). Iron content (PIXE) in competent and incompetent veins is related to the vein wall morphology and tissue antioxidant enzymes. Bioelectrochemistry.

[105] Jomova K., Alomar S.Y., Alwasel S.H., Nepovimova E., Kuca K., Valko M. (2024). Several lines of antioxidant defense against oxidative stress: Antioxidant enzymes, nanomaterials with multiple enzyme-mimicking activities, and low-molecular-weight antioxidants. Arch. Toxicol..

[106] Khelfi A., Andreescu S., Henkel R., Khelfi A. (2024). Antioxidants. Biomarkers of Oxidative Stress: Basics and Measurement of Oxidative Stress.

[107] Halliwell B., Gutteridge J.M. (1995). The definition and measurement of antioxidants in biological systems. Free Radic. Biol. Med..

[108] Silvestrini A., Mancini A. (2024). The Double-Edged Sword of Total Antioxidant Capacity: Clinical Significance and Personal Experience. Antioxidants.

[109] Horecka A., Biernacka J., Hordyjewska A., Dąbrowski W., Terlecki P., Zubilewicz T., Musik I., Kurzepa J. (2018). Antioxidative mechanism in the course of varicose veins. Phlebology.

[110] Birben E., Sahiner U.M., Sackesen C., Erzurum S., Kalayci O. (2012). Oxidative Stress and Antioxidant Defense. World Allergy Organ. J..

[111] Forman H.J., Zhang H., Rinna A. (2009). Glutathione: Overview of its protective roles, measurement, and biosynthesis. Mol. Asp. Med..

[112] Mirończuk-Chodakowska I., Witkowska A.M., Zujko M.E. (2018). Endogenous non-enzymatic antioxidants in the human body. Adv. Med. Sci..

[113] Wali M.A., Suleiman S.A., Kadoumi O.F., Nasr M.A. (2002). Superoxide radical concentration and superoxide dismutase (SOD) enzyme activity in varicose veins. Ann. Thorac. Cardiovasc. Surg..

[114] Krzyściak W., Cierniak A., Kózka M., Kozieł J. (2011). Oxidative DNA Damage in Blood of CVD Patients Taking Detralex. Open Cardiovasc. Med. J..

[115] Krzyściak W., Kózka M., Kowalska J., Kwiatek W.M. (2010). Role of Zn, Cu--trace elements and superoxide dismutase (SOD) in oxidative stress progression in chronic venous insufficiency (CVI). Przegląd Lek..

[116] Triankina S.A., Kolobova O.I., Varshavskiĭ B. (2003). The role of peroxidation in pathogenesis of varicose veins. Klin. Lab. Diagn..

[117] Modaghegh M.H.S., Saberianpour S., Amoueian S., Kamyar M.M. (2022). Signaling pathways associated with structural changes in varicose veins: A case-control study. Phlebology.

[118] Vašková J., Kočan L., Vaško L., Perjési P. (2023). Glutathione-Related Enzymes and Proteins: A Review. Molecules.

[119] Prasad A., Andrews N.P., Padder F.A., Husain M., Quyyumi A.A. (1999). Glutathione reverses endothelial dysfunction and improves nitric oxide bioavailability. J. Am. Coll. Cardiol..

[120] Rafea R., Siragusa M., Fleming I. (2025). The Ever-Expanding Influence of the Endothelial Nitric Oxide Synthase. Basic. Clin. Pharmacol. Toxicol..

[121] Aucoin M.M., Barhoumi R., Kochevar D.T., Granger H.J., Burghardt R.C. (1995). Oxidative injury of coronary venular endothelial cells depletes intracellular glutathione and induces HSP 70 mRNA. Am. J. Physiol..

[122] Noguchi N., Saito Y., Niki E. (2023). Actions of Thiols, Persulfides, and Polysulfides as Free Radical Scavenging Antioxidants. Antioxid. Redox Signal.

[123] Trujillo M., Alvarez B., Radi R. (2016). One- and two-electron oxidation of thiols: Mechanisms, kinetics and biological fates. Free Radic. Res..

[124] Poredos P., Spirkoska A., Rucigaj T., Fareed J., Jezovnik M.K. (2015). Do Blood Constituents in Varicose Veins Differ From the Systemic Blood Constituents?. Eur. J. Vasc. Endovasc. Surg..

[125] Turell L., Radi R., Alvarez B. (2013). The thiol pool in human plasma: The central contribution of albumin to redox processes. Free Radic. Biol. Med..

[126] Belinskaia D.A., Voronina P.A., Shmurak V.I., Vovk M.A., Batalova A.A., Jenkins R.O., Goncharov N.V. (2020). The Universal Soldier: Enzymatic and Non-Enzymatic Antioxidant Functions of Serum Albumin. Antioxidants.

[127] Sautin Y.Y., Johnson R.J. (2008). Uric acid: The oxidant-antioxidant paradox. Nucleosides Nucleotides Nucleic Acids.

[128] Albuja-Quintana N., Chisaguano-Tonato A.M., Herrera-Fontana M.E., Figueroa-Samaniego S., Alvarez-Suarez J.M. (2025). Relationship between plasma uric acid levels, antioxidant capacity, and oxidative damage markers in overweight and obese adults: A cross-sectional study. PLoS ONE.

[129] Xu L., Li C., Wan T., Sun X., Lin X., Yan D., Li J., Wei P. (2025). Targeting uric acid: A promising intervention against oxidative stress and neuroinflammation in neurodegenerative diseases. Cell Commun. Signal..

[130] Michiels C., Arnould T., Remacle J. (1993). Hypoxia-Induced Activation of Endothelial Cells as a Possible Cause of Venous Diseases: Hypothesis. Angiology.

[131] Michiels C., Arnould T., Houbion A., Remacle J. (1992). Human umbilical vein endothelial cells submitted to hypoxia-reoxygenation in vitro: Implication of free radicals, xanthine oxidase, and energy deficiency. J. Cell Physiol..

[132] Waring W.S., Webb D.J., Maxwell S.R. (2001). Systemic uric acid administration increases serum antioxidant capacity in healthy volunteers. J. Cardiovasc. Pharmacol..

[133] Gęgotek A., Skrzydlewska E. (2023). Ascorbic acid as antioxidant. Vitam. Horm..

[134] Murphy M.P., Bayir H., Belousov V., Chang C.J., Davies K.J.A., Davies M.J., Dick T.P., Finkel T., Forman H.J., Janssen-Heininger Y. (2022). Guidelines for measuring reactive oxygen species and oxidative damage in cells and in vivo. Nat. Metab..

[135] Giustarini D., Tsikas D., Colombo G., Milzani A., Dalle-Donne I., Fanti P., Rossi R. (2016). Pitfalls in the analysis of the physiological antioxidant glutathione (GSH) and its disulfide (GSSG) in biological samples: An elephant in the room. J. Chromatogr. B Anal. Technol. Biomed. Life Sci..

[136] Sies H., Belousov V.V., Chandel N.S., Davies M.J., Jones D.P., Mann G.E., Murphy M.P., Yamamoto M., Winterbourn C. (2022). Defining roles of specific reactive oxygen species (ROS) in cell biology and physiology. Nat. Rev. Mol. Cell Biol..

[137] Deshwal S., Antonucci S., Kaludercic N., Di Lisa F. (2018). Measurement of Mitochondrial ROS Formation. Methods Mol. Biol..

[138] Guzik T.J., Harrison D.G. (2006). Vascular NADPH oxidases as drug targets for novel antioxidant strategies. Drug Discov. Today.

[139] Kawagishi H., Finkel T. (2014). Unraveling the Truth About Antioxidants: ROS and disease: Finding the right balance. Nat. Med..

[140] Nitti M., Marengo B., Furfaro A.L., Pronzato M.A., Marinari U.M., Domenicotti C., Traverso N. (2022). Hormesis and Oxidative Distress: Pathophysiology of Reactive Oxygen Species and the Open Question of Antioxidant Modulation and Supplementation. Antioxidants.

[141] Lassègue B., San Martín A., Griendling K.K. (2012). Biochemistry, physiology, and pathophysiology of NADPH oxidases in the cardiovascular system. Circ. Res..

[142] Li Y., Pagano P.J. (2017). Microvascular NADPH oxidase in health and disease. Free Radic. Biol. Med..

[143] Zhang Y., Murugesan P., Huang K., Cai H. (2020). NADPH oxidases and oxidase crosstalk in cardiovascular diseases: Novel therapeutic targets. Nat. Rev. Cardiol..

[144] Sirker A., Zhang M., Shah A.M. (2011). NADPH oxidases in cardiovascular disease: Insights from in vivo models and clinical studies. Basic. Res. Cardiol..

[145] La Favor J.D., Dubis G.S., Yan H., White J.D., Nelson M.A., Anderson E.J., Hickner R.C. (2016). Microvascular Endothelial Dysfunction in Sedentary, Obese Humans Is Mediated by NADPH Oxidase: Influence of Exercise Training. Arter. Thromb. Vasc. Biol..

[146] Guzik T.J., West N.E.J., Black E., McDonald D., Ratnatunga C., Pillai R., Channon K.M. (2000). Vascular Superoxide Production by NAD(P)H Oxidase. Circ. Res..

[147] McNally J.S., Davis M.E., Giddens D.P., Saha A., Hwang J., Dikalov S., Jo H., Harrison D.G. (2003). Role of xanthine oxidoreductase and NAD(P)H oxidase in endothelial superoxide production in response to oscillatory shear stress. Am. J. Physiol. Heart Circ. Physiol..

[148] Kirkman D.L., Robinson A.T., Rossman M.J., Seals D.R., Edwards D.G. (2021). Mitochondrial contributions to vascular endothelial dysfunction, arterial stiffness, and cardiovascular diseases. Am. J. Physiol. Heart Circ. Physiol..

[149] Kluge M.A., Fetterman J.L., Vita J.A. (2013). Mitochondria and endothelial function. Circ. Res..

[150] Zorov D.B., Juhaszova M., Sollott S.J. (2014). Mitochondrial reactive oxygen species (ROS) and ROS-induced ROS release. Physiol. Rev..

[151] Zinkevich N.S., Gutterman D.D. (2011). ROS-induced ROS release in vascular biology: Redox-redox signaling. Am. J. Physiol. Heart Circ. Physiol..

[152] Madamanchi N.R., Vendrov A., Runge M.S. (2005). Oxidative stress and vascular disease. Arter. Thromb. Vasc. Biol..

[153] Batty M., Bennett M.R., Yu E. (2022). The Role of Oxidative Stress in Atherosclerosis. Cells.

[154] Craige S.M., Kaur G., Bond J.M., Caliz A.D., Kant S., Keaney J.F. (2025). Endothelial Reactive Oxygen Species: Key Players in Cardiovascular Health and Disease. Antioxid. Redox Signal.

[155] Sies H. (2024). Dynamics of intracellular and intercellular redox communication. Free Radic. Biol. Med..

[156] Crowley S.D. (2014). The cooperative roles of inflammation and oxidative stress in the pathogenesis of hypertension. Antioxid. Redox Signal.

[157] Maruhashi T., Higashi Y. (2021). Pathophysiological Association between Diabetes Mellitus and Endothelial Dysfunction. Antioxidants.

[158] Forrester S.J., Kikuchi D.S., Hernandes M.S., Xu Q., Griendling K.K. (2018). Reactive Oxygen Species in Metabolic and Inflammatory Signaling. Circ. Res..

[159] Seyedsadjadi N., Grant R. (2020). The Potential Benefit of Monitoring Oxidative Stress and Inflammation in the Prevention of Non-Communicable Diseases (NCDs). Antioxidants.

[160] Peña-Oyarzun D., Bravo-Sagua R., Diaz-Vega A., Aleman L., Chiong M., Garcia L., Bambs C., Troncoso R., Cifuentes M., Morselli E. (2018). Autophagy and oxidative stress in non-communicable diseases: A matter of the inflammatory state?. Free Radic. Biol. Med..

[161] Wang M., Xiao Y., Miao J., Zhang X., Liu M., Zhu L., Liu H., Shen X., Wang J., Xie B. (2025). Oxidative Stress and Inflammation: Drivers of Tumorigenesis and Therapeutic Opportunities. Antioxidants.

[162] Biswas S.K. (2016). Does the Interdependence between Oxidative Stress and Inflammation Explain the Antioxidant Paradox?. Oxid. Med. Cell Longev..

[163] Hulsmans M., Holvoet P. (2010). The vicious circle between oxidative stress and inflammation in atherosclerosis. J. Cell Mol. Med..

[164] Petrascu F.-M., Matei S.-C., Margan M.-M., Ungureanu A.-M., Olteanu G.-E., Murariu M.-S., Olariu S., Marian C. (2024). The Impact of Inflammatory Markers and Obesity in Chronic Venous Disease. Biomedicines.

[165] Wadley A.J., van Zanten J.J.C.S.V., Aldred S. (2013). The interactions of oxidative stress and inflammation with vascular dysfunction in ageing: The vascular health triad. AGE.

[166] Ortega M.A., Asúnsolo Á., Leal J., Romero B., Alvarez-Rocha M.J., Sainz F., Álvarez-Mon M., Buján J., García-Honduvilla N. (2018). Implication of the PI3K/Akt/mTOR Pathway in the Process of Incompetent Valves in Patients with Chronic Venous Insufficiency and the Relationship with Aging. Oxid. Med. Cell Longev..

[167] Vona R., Pallotta L., Cappelletti M., Severi C., Matarrese P. (2021). The Impact of Oxidative Stress in Human Pathology: Focus on Gastrointestinal Disorders. Antioxidants.

[168] Raffetto J.D., Khalil R.A. (2021). Mechanisms of Lower Extremity Vein Dysfunction in Chronic Venous Disease and Implications in Management of Varicose Veins. Vessel. Plus.

[169] di Candia A.M., de Avila D.X., Moreira G.R., Villacorta H., Maisel A.S. (2021). Growth differentiation factor-15, a novel systemic biomarker of oxidative stress, inflammation, and cellular aging: Potential role in cardiovascular diseases. Am. Heart J. Plus: Cardiol. Res. Pract..

[170] Sreelakshmi B.J., Karthika C.L., Ahalya S., Kalpana S.R., Kartha C.C., Sumi S. (2024). Mechanoresponsive ETS1 causes endothelial dysfunction and arterialization in varicose veins via NOTCH4/DLL4 signaling. Eur. J. Cell Biol..

[171] Chandran Latha K., Sreekumar A., Beena V., S.S. B.R., Lakkappa R.B., Kalyani R., Nair R., Kalpana S.R., Kartha C.C., Surendran S. (2021). Shear Stress Alterations Activate BMP4/pSMAD5 Signaling and Induce Endothelial Mesenchymal Transition in Varicose Veins. Cells.

[172] Serralheiro P., Soares A., Costa Almeida C.M., Verde I. (2017). TGF-β1 in Vascular Wall Pathology: Unraveling Chronic Venous Insufficiency Pathophysiology. Int. J. Mol. Sci..

[173] Li S., Liu Y., Liu M., Wang L., Li X. (2022). Comprehensive bioinformatics analysis reveals biomarkers of DNA methylation-related genes in varicose veins. Front. Genet..

[174] Fukaya E., Flores A.M., Lindholm D., Gustafsson S., Zanetti D., Ingelsson E., Leeper N.J. (2018). Clinical and Genetic Determinants of Varicose Veins. Circulation.

[175] Jones D.P., Sies H. (2015). The Redox Code. Antioxid. Redox Signal.

[176] Jin S., Kang P.M. (2024). A Systematic Review on Advances in Management of Oxidative Stress-Associated Cardiovascular Diseases. Antioxidants.

[177] Bielli A., Scioli M.G., Mazzaglia D., Doldo E., Orlandi A. (2015). Antioxidants and vascular health. Life Sci..

[178] Mostafa R.E., Ali D.E., El-Shiekh R.A., El-Alfy A.N., Hafeez M., Reda A.M., Fayek N.M. (2025). Therapeutic applications of natural products in the management of venous diseases: A comprehensive review. Inflammopharmacology.

[179] Dimauro I., Paronetto M.P., Caporossi D. (2020). Exercise, redox homeostasis and the epigenetic landscape. Redox Biol..

[180] Lichota A., Gwozdzinski L., Gwozdzinski K. (2019). Therapeutic potential of natural compounds in inflammation and chronic venous insufficiency. Eur. J. Med. Chem..

[181] Bencsik T., Balázs V.L., Farkas Á., Csikós E., Horváth A., Ács K., Kocsis M., Doseděl M., Fialová S.B., Czigle S. (2024). Herbal drugs in chronic venous disease treatment: An update. Fitoterapia.

[182] Casili G., Lanza M., Campolo M., Messina S., Scuderi S., Ardizzone A., Filippone A., Paterniti I., Cuzzocrea S., Esposito E. (2021). Therapeutic potential of flavonoids in the treatment of chronic venous insufficiency. Vasc. Pharmacol..

[183] Diaz J.A., Gianesini S., Khalil R.A. (2024). Glycocalyx disruption, endothelial dysfunction and vascular remodeling as underlying mechanisms and treatment targets of chronic venous disease. Int. Angiol..

[184] Mansilha A., Sousa J. (2018). Pathophysiological Mechanisms of Chronic Venous Disease and Implications for Venoactive Drug Therapy. Int. J. Mol. Sci..

[185] Bjørklund G., Chirumbolo S. (2017). Role of oxidative stress and antioxidants in daily nutrition and human health. Nutrition.

[186] Zhang Y.J., Gan R.Y., Li S., Zhou Y., Li A.N., Xu D.P., Li H.B. (2015). Antioxidant Phytochemicals for the Prevention and Treatment of Chronic Diseases. Molecules.

[187] Salah H.M., Verma S., Santos-Gallego C.G., Bhatt A.S., Vaduganathan M., Khan M.S., Lopes R.D., Al’Aref S.J., McGuire D.K., Fudim M. (2022). Sodium-Glucose Cotransporter 2 Inhibitors and Cardiac Remodeling. J. Cardiovasc. Transl. Res..

[188] Santos G.L., Dos Santos C.F., Rocha G.R., Calmon M.S., Lemos F.F., Silva L.G., Luz M.S., Pinheiro S.L., Botelho A.C., de Melo F.F. (2025). Beyond glycemic control: Roles for sodium-glucose cotransporter 2 inhibitors and glucagon-like peptide-1 receptor agonists in diabetic kidney disease. World J. Diabetes.

[189] Sanz R.L., Menéndez S.G., Inserra F., Ferder L., Manucha W. (2024). Cellular and Mitochondrial Pathways Contribute to SGLT2 Inhibitors-mediated Tissue Protection: Experimental and Clinical Data. Curr. Pharm. Des..

[190] Ionică L.N., Lința A.V., Bătrîn A.D., Hâncu I.M., Lolescu B.M., Dănilă M.D., Petrescu L., Mozoș I.M., Sturza A., Muntean D.M. (2024). The off-Target Cardioprotective Mechanisms of Sodium-Glucose Cotransporter 2 Inhibitors: An Overview. Int. J. Mol. Sci..

[191] Patel T.A., Zheng H., Patel K.P. (2025). Sodium–Glucose Cotransporter 2 Inhibitors as Potential Antioxidant Therapeutic Agents in Cardiovascular and Renal Diseases. Antioxidants.

[192] Mroueh A., Algara-Suarez P., Fakih W., Gong D.S., Matsushita K., Park S.H., Amissi S., Auger C., Kauffenstein G., Meyer N. (2025). SGLT2 expression in human vasculature and heart correlates with low-grade inflammation and causes eNOS-NO/ROS imbalance. Cardiovasc. Res..

[193] Ionică L.N., Buriman D.G., Lința A.V., Șoșdean R., Lascu A., Streian C.G., Feier H.B., Petrescu L., Mozoș I.M., Sturza A. (2025). Empagliflozin and dapagliflozin decreased atrial monoamine oxidase expression and alleviated oxidative stress in overweight non-diabetic cardiac patients. Mol. Cell Biochem..

[194] Beucher L., Gabillard-Lefort C., Baris O.R., Mialet-Perez J. (2024). Monoamine oxidases: A missing link between mitochondria and inflammation in chronic diseases?. Redox Biol..

[195] Robinson D.S., Nies A., Davis J.N., Bunney W.E., Davis J.M., Colburn R.W., Bourne H.R., Shaw D.M., Coppen A.J. (1972). Ageing, Monoamines, And Monoamine-Oxidase Levels. Lancet.

[196] Maggiorani D., Manzella N., Edmondson D.E., Mattevi A., Parini A., Binda C., Mialet-Perez J. (2017). Monoamine Oxidases, Oxidative Stress, and Altered Mitochondrial Dynamics in Cardiac Ageing. Oxid. Med. Cell Longev..

[197] Hess D.A., Verma S., Bhatt D., Bakbak E., Terenzi D.C., Puar P., Cosentino F. (2022). Vascular repair and regeneration in cardiometabolic diseases. Eur. Heart J..

[198] Terenzi D.C., Bakbak E., Teoh H., Krishnaraj A., Puar P., Rotstein O.D., Cosentino F., Goldenberg R.M., Verma S., Hess D.A. (2024). Restoration of blood vessel regeneration in the era of combination SGLT2i and GLP-1RA therapy for diabetes and obesity. Cardiovasc. Res..

[199] Mameli E., Martello A., Caporali A. (2022). Autophagy at the interface of endothelial cell homeostasis and vascular disease. FEBS J..

[200] Hu M., Ladowski J.M., Xu H. (2024). The Role of Autophagy in Vascular Endothelial Cell Health and Physiology. Cells.

[201] Ren H., Dai R., Nik Nabil W.N., Xi Z., Wang F., Xu H. (2023). Unveiling the dual role of autophagy in vascular remodelling and its related diseases. Biomed. Pharmacother..

[202] Okazaki R.A., Rizvi S.H., Lyons R., Behrooz L., Hamburg N.M. (2025). Vascular Dysfunction in Diabetes and Pharmacotherapeutic Opportunities: A Focus on Endothelial Cell Health. Am. J. Physiol. Heart Circ. Physiol..

[203] Larionov A., Hammer C.M., Fiedler K., Filgueira L. (2024). Dynamics of Endothelial Cell Diversity and Plasticity in Health and Disease. Cells.

[204] Zhong J., Gao R.R., Zhang X., Yang J.X., Liu Y., Ma J., Chen Q. (2025). Dissecting endothelial cell heterogeneity with new tools. Cell Regen..

[205] Zhang B., Schmidlin T. (2024). Recent advances in cardiovascular disease research driven by metabolomics technologies in the context of systems biology. npj Metab. Health Dis..

